# Selective Conversion of Polyolefin Waste to Branched Alkanes via Methane‐Free Tandem Hydrocracking–Isomerization

**DOI:** 10.1002/advs.202516707

**Published:** 2026-02-03

**Authors:** Xinbang Wu, Sitan Wang, Matilde Onofri, Kande Liu, Kun‐Han Lin, Roland C. Turnell‐Ritson, Li Shi, Xuan Meng, Paul J. Dyson

**Affiliations:** ^1^ Institute of Chemical Sciences and Engineering École Polytechnique Fédérale de Lausanne (EPFL) Lausanne Switzerland; ^2^ The State Key Laboratory of Chemical Engineering East China University of Science and Technology Shanghai China; ^3^ Department of Chemical Engineering National Tsing Hua University Hsinchu Taiwan; ^4^ Chemistry Research Laboratory Department of Chemistry University of Oxford Oxford UK

**Keywords:** heterogeneous catalysis, hydrocracking, plastic upcycling

## Abstract

In recent years, catalytic hydrocracking has emerged as a promising waste‐to‐feedstock solution for processing plastic waste. Although ruthenium nanoparticle catalysts exhibit high activity in converting polyolefins into small alkanes, they also tend to produce undesirable methane (CH_4_) via terminal C─C bond hydrogenolysis. Herein, we report a Ru‐supported sulfated zirconia (RuSZ_1_) catalyst for the efficient isomerization and hydrocracking of polyolefins without generating CH_4_. Various high‐M_w_ polyethylene and polypropylene samples (up to 1 000 000 g/mol) were fully converted into branched alkanes (C_4_‐C_20_). Control experiments using RuSZ_1_ revealed negligible scission activity for alkanes with fewer than seven carbons, suggesting that isomerization precedes hydrocracking. Hydrogenolysis to form C_1_ and C_2_ products is likely inhibited by saturation of sulfate species at the Ru sites, and these products were not observed under any reaction conditions. Full conversion of polyolefins under solvent‐free conditions was achieved, with up to 93% yield of C_3_‐C_12_ alkanes.

## Graphical abstract

Scheme illustrating the selective conversion of plastic waste to isomerized C4‐C12 products using RuSZ1 under different reaction conditions.

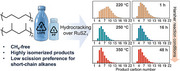

## 1 Introduction

**SCHEME 1:**
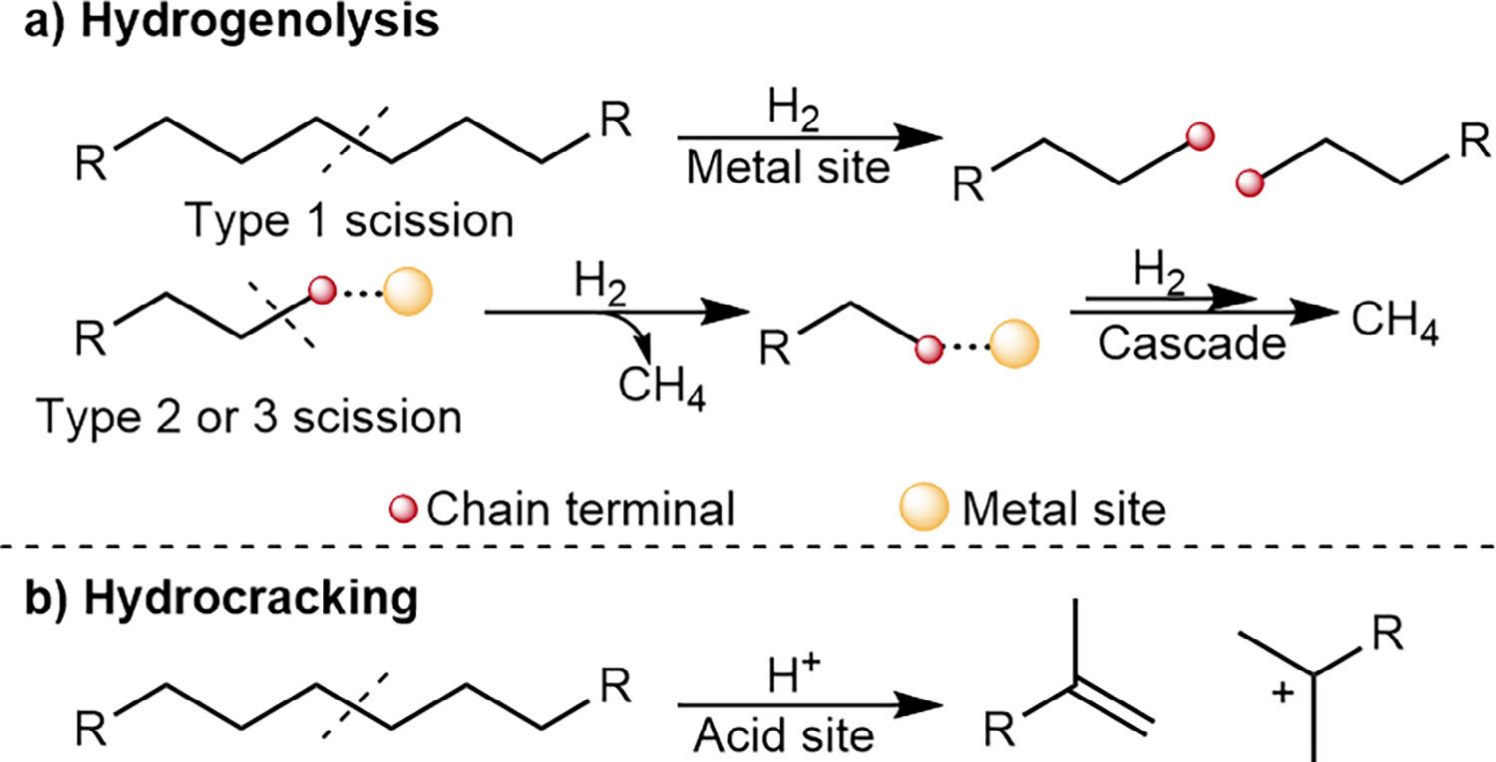
Different C─C bond cleaving mechanisms. (a) Metal site‐mediated hydrogenolysis and (b) acid site‐mediated hydrocracking of C─C bonds.

The generation and mismanagement of plastic waste continues on an upward trend [1], with the improper disposal of, in particular, single‐use plastics predominantly consisting of polyolefins, leading to serious environmental and health impacts [2]. Polyethylene (PE) and polypropylene (PP) are the two most common plastics, together accounting for more than 45% of the total market [3]. The majority of PE and PP waste is processed through incineration or landfilling, which is uneconomical given that these polymers are sourced from finite fossil fuels [4]. In recent years, the depolymerization of waste polyolefins using heterogeneous [5, 6, 7, 8, 9, 10, 11] and homogeneous [12, 13, 14] catalysts has been demonstrated to be a viable pathway to produce short‐chain hydrocarbons suitable for industrial or fuel applications. Heterogeneous processes typically utilize bifunctional catalysts, consisting of an active noble metal deposited on an acidic support, to selectively convert polyolefins into liquid products under pressurized hydrogen (H_2_) [15, 16, 17, 18, 19, 20, 21, 22, 23, 24, 25, 26]. Under depolymerization conditions, the acid sites promote the cracking of the carbon‐carbon (C─C) bonds within the polymer, while the metal sites facilitate hydrogenation of the reaction intermediates while mitigating coke formation [27, 28]. In addition, the metal sites also promote hydrogenolysis, where C─C bonds are directly cleaved using H_2_ to generate two new chain‐ends (Scheme 1, Type 1 scission) [27]. However, hydrogenolysis typically occurs at the chain terminal of the polymer or adsorbed intermediates (Types 2 and 3 scission) [24], producing methane (CH_4_), which is less valuable than liquid products due to lower chemical complexity and higher stoichiometric H_2_ consumption. Despite achieving significantly faster rates compared to other metal catalysts [28, 29, 30], Ru catalysts are particularly prone to generating CH_4_ during polyolefin conversion, as the Ru(0001) surface is highly active for C─C bond hydrogenolysis [31].

Compared to gaseous alkanes, liquid alkanes (C_5_‐C_20_) are more valuable as fuels due to their higher energy density, which could be used as gasoline (C_5_‐C_12_) or diesel (C_13_‐C_20_). In addition, they may also be used as an alternative feedstock to fossil‐based hydrocarbons. To date, a major challenge for polyolefin upcycling with Ru catalysts has been balancing the selectivity between liquid and gas products, since both are usually formed at high polymer conversions (Table 1). Alloying the Ru sites with a less active metal, such as Ni or Pt, is a potential strategy to increase the selectivity to liquid alkanes while maintaining high conversion rates [32, 33]. Alternatively, the catalyst can be modified to partially suppress CH_4_ formation at either the Ru sites or the support. Smaller Ru sites (< 2 nm) have been reported to inhibit the adsorption of alkane intermediates, which avoids Type 3 scission after hydrocracking [19, 22]. ZrO_2_‐supported Ru nanoparticles of around 2.5 nm can achieve up to 79% selectivity to liquid alkanes, although CH_4_ remains a major product [23]. The yield of CH_4_ decreases further when the ZrO_2_ support is doped with reducible oxides, which promotes reverse H_2_ spillover to the Ru sites, enabling the rapid hydrogenation of intermediates following Type 1 scission [24, 25].

**TABLE 1:** Strategies employed with Ru‐supported catalysts to target liquid alkanes from PE.

Entry	Ru / (support)	Temperature (°C)	H_2_ pressure (bar)	Time (h)	C₁ yield (%)	Gaseous product yield (%)	Liquid product yield (%)	Reference
1	FAU zeolite	300	50	3	68	96	4	15
2	FAU zeolite	200	30	16	NA	33^a^	67^b^	16
3	C	200	22	16	32	58	42	17
4	CeO_2_	260	30	3	18	NA	47	18
5	CeO_2_	240	20	4	NA	29	57	19
6	TiO_2_–anatase	225	20	4	9	29^a^	34^c^	20
7	TiO_2_–rutile	200	30	3	NA	9	61	21
8	MgAl‐LDO	240	40	4	2	6	72	22
9	ZrO_2_	200	20	10	NA	15	79	23
10	W‐doped ZrO_2_	250	50	2	9	NA	63^d^	24
11	WO_3_–ZrO_2_	250	50	2	5	7	28^e^	25
12	ZrO_2_ ^f^	250	30	8	0	28^g^	69	26
**13**	**Sulfated‐ZrO_2_**	**220**	**30**	**2**	**0**	**9**	**87** ^e^	**This work**

Gaseous and liquid alkanes represent C_1_‐C_4_ and C_5_‐C_20_, respectively, unless otherwise stated.

^a^ C_1_‐C_5_;

^b^ C_6_‐C_32_;

^c^ C_6_‐C_45_;

^d^ C_4_‐C_35;_

^e^ C_5_‐C_12_;

^f^ Ru single‐atoms;

^g^ includes isomerized C_5_‐C_7_. NA: data not available.

Another strategy to mitigate CH_4_ formation is to engage hydrocracking as the only C─C bond cleavage pathway. Selectivity can be tuned toward longer chain alkanes by restricting hydrogenolysis at the metal sites, and mainly utilizing these sites for the initial reaction activation and the hydrogenation of cracking intermediates [7]. This approach requires a catalyst with a high acid‐to‐metal ratio, which suppresses hydrogenolysis while enhancing hydrogenation of intermediates through metal‐support interactions [34, 35]. Furthermore, the strong acid sites also facilitate isomerization of the alkane products through rearrangement of linear carbenium ions after hydrocracking [36], which is beneficial for improving the octane rating and viscosity of the products for fuel applications [37]. Pt supported on sulfated‐ZrO_2_ (SZ) catalysts have been explored for the tandem hydrocracking‐isomerization of PE above 325°C, achieving up to 64% yield of liquid alkanes with an iso‐/*n‐*alkane factor of around 24 [38]. Due to the low cost and high Brønsted acid site density of SZ, which is produced from the treatment of ZrO_2_ with sulfuric acid (H_2_SO_4_), it has been extensively studied as a catalyst for various processes, including isomerization, alkylation, esterification, and cracking [39]. Hence, investigating Ru‐supported SZ catalysts presents a promising yet underexplored opportunity for polyolefin upcycling into highly isomerized alkanes, given the intrinsically higher depolymerization ability of Ru compared to other metals.

Herein, we report a catalyst consisting of Ru nanoparticles supported on SZ (RuSZ_1_) for the conversion of polyolefins to liquid alkanes in excellent yield (up to 91%), with an iso‐/*n‐*alkane factor of 8.5 for the conversion of PE. Notably, the catalyst effectively suppresses the formation of C_1_ and C_2_ products while maintaining high selectivity toward liquid fuels. The rate of conversion is directly correlated with the carbon chain length of the reactant, and the catalyst exhibits low scission preference for reactants containing fewer than seven carbons. Various commercially available polyolefin products were fully converted to branched liquid alkanes, demonstrating a potential waste‐to‐fuel pathway. A preliminary cost analysis reveals that the cost of gasoline generated from the plastic waste‐to‐fuel pathway will be 72% less expensive compared to hydrocracking of vacuum gas oil.

## 2 Results and Discussion

### 2.1 Synthesis and Characterization of RuSZ_x_ Catalysts

**FIGURE 1:**
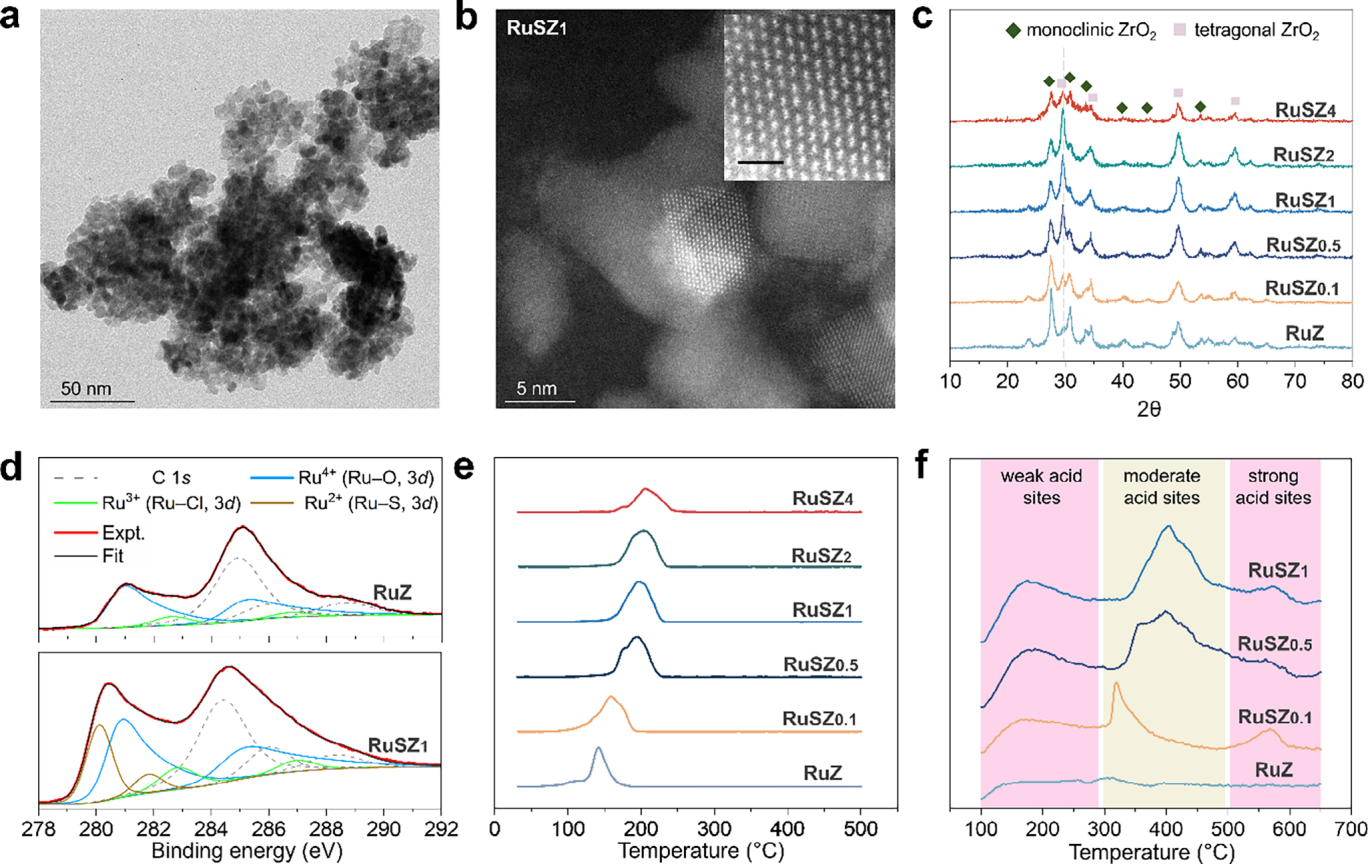
Characterization of the catalysts. (a) TEM image of the non‐sulfated ZrO_2_ support. (b) HR‐STEM of RuSZ_1_, inset showing the magnified region of a Ru nanoparticle, scale bar 1 nm. (c) PXRD spectra revealing the evolution of the peak at 30° with increasing degree of sulfation of RuSZ_x_. (d) XPS comparing the different Ru coordination environments between RuZ and RuSZ_1_. (e) H_2_‐TPR profiles showing the reduction temperatures of the RuSZ_x_ catalysts. (f) NH_3_‐TPD profiles correlating the degree of sulfation with the amount of surface acid sites.

As the ratio of acid‐to‐metal sites on a catalyst influences both the isomerization and hydrocracking mechanism [5, 7], Ru (5 wt. %) supported on SZ*_x_* catalysts was synthesized with differing degrees of sulfation for screening. The degree of sulfation in SZ*_x_* was controlled by treating the Zr(OH)_4_ precursor with different H_2_SO_4_ concentrations (where *x* = 0, 0.1, 0.5, 1, 2 and 4 M). Ru was loaded onto SZ*_x_* via wet‐impregnation and the catalysts were calcined in air at 550°C (see Methods section for detailed procedure of catalyst synthesis and Tables S1 and S2 for the sulfur and Ru content in each catalyst). Transmission electron microscopy (TEM) images of the support without sulfation reveal small ZrO_2_ nanoparticles with an average diameter of ∼9 nm (Figure 1a; see Figure S1a for particle size distribution). Observation of RuSZ_1_ with high‐resolution scanning transmission electron microscopy (HR‐STEM) reveals that the size of the ZrO_2_ support remains unchanged after sulfation, and that the Ru species comprise nanoparticles of ∼3 nm (Figure 1b; see Figures S1b for particle size distribution and S2 for EDX mapping). The non‐sulfated catalyst, RuZ, exhibits both monoclinic and tetragonal phases, as determined by powder X‐ray diffraction (PXRD; Figure 1c). As the degree of sulfation increases, the tetragonal phase of ZrO_2_ becomes more stabilized, evidenced by the evolution of the peak at 30 °, which is directly correlated to the isomerization activity of the catalyst [40]. However, due to the heavy sulfation in RuSZ_4_, the broadening of the PXRD peaks reveals that the support degrades into a more amorphous phase. Due to the low Ru loading and small Ru nanoparticle size, Ru peaks were not detected from the PXRD spectra of the catalysts.

The coordination environments of RuZ and RuSZ_1_ were analyzed using X‐ray photoelectron spectroscopy (XPS; Figure 1d), revealing that Ru is primarily coordinated to oxygen as Ru(IV), with small amounts of Ru(III) species also present. Additional peaks at 280.1 and 281.9 eV were observed from the spectra of RuSZ_1_, which may be ascribed to coordination of Ru(II) to sulfur species [41, 42]. Although only cationic Ru^δ+^ species were detected from XPS analysis, these species would be reduced in situ during polyolefin conversion in H_2_ [43]. Hence, H_2_ temperature‐programmed reduction (H_2_‐TPR) was conducted to determine the reduction temperature of the RuSZ*_x_* catalysts (Figure 1e). The results reveal that a higher degree of sulfation leads to an increased reduction temperature of the catalyst. This is likely due to more Ru(II) species present in the more sulfated catalysts. RuZ has an H_2_ consumption maximum at 141°C, whereas RuSZ_0.1_ and RuSZ_0.5_ display maxima at 158°C and 195°C, respectively. However, there is a negligible increase in reduction temperature at sulfation levels beyond RuSZ_1_ (maximum at 200°C), likely due to saturation of surface sulfate species. RuZ and RuSZ*_x_* (*x* = 0.1–1) were further studied by NH_3_ temperature programmed desorption (NH_3_‐TPD) to compare their available surface acid sites (Figure 1f). RuZ has negligible acidic sites, whereas the RuSZ*_x_* catalysts show increasing amounts of weak and moderate acid sites, which are crucial for promoting cracking and isomerization, with a higher degree of sulfation [39].

### 2.2 Effect of Sulfation on Catalyst Activity and Selectivity

**FIGURE 2:**
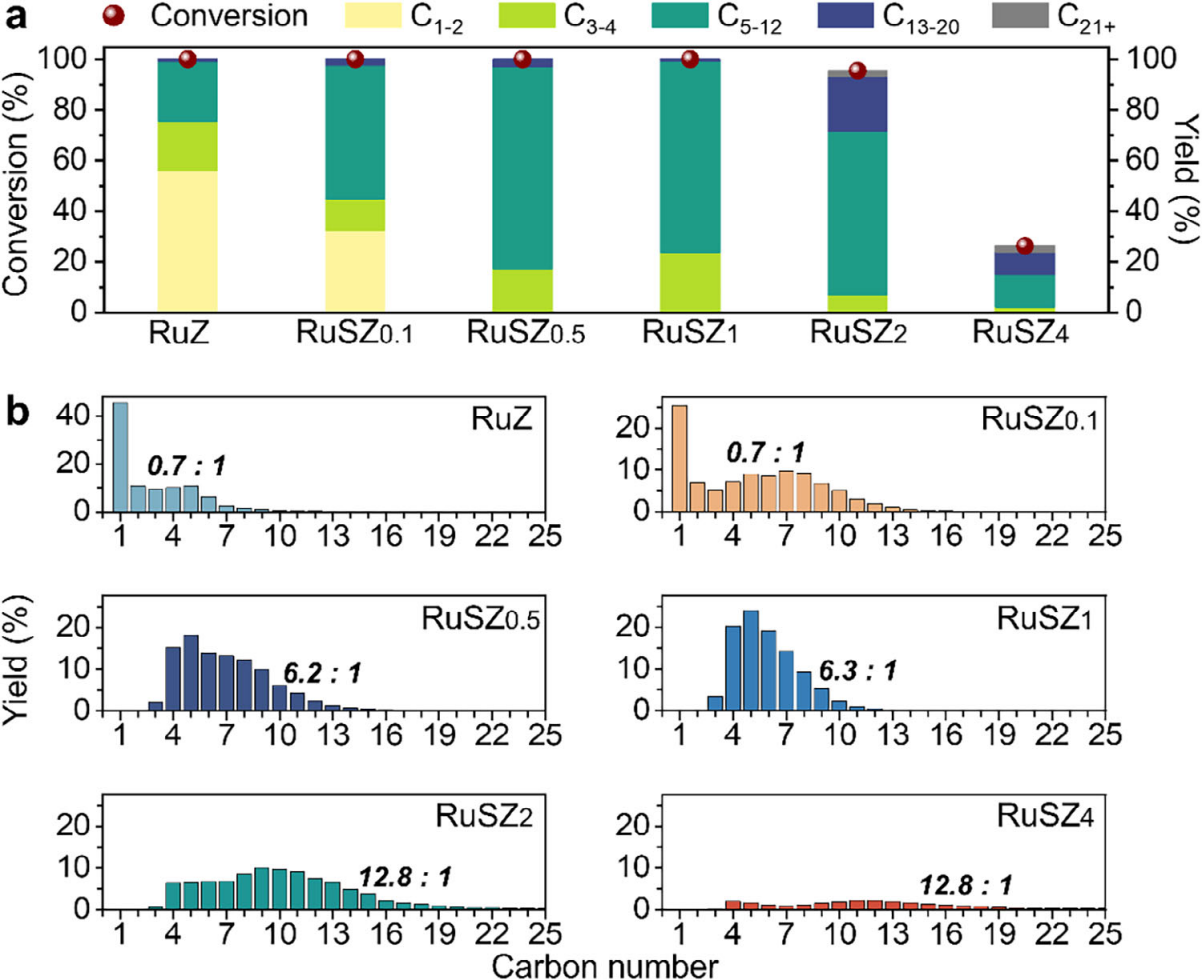
Effect of the degree of sulfation of RuSZ*_x_* on the catalyst activity and product selectivity for the hydrocracking of LDPE. (a) Conversion and product yields. (b) Product yield distributions with iso‐/*n‐*alkane ratios are shown in bold italics. Yields of all the products are available in Table S3. Reaction conditions: 500 mg LDPE, 50 mg catalyst (0.5 wt. % Ru), 250°C, 30 bar H_2_, 4 h.

The catalysts were screened using low‐density PE (LDPE powder, M_w_ = 4000 g/mol) to assess the influence of the degree of catalyst support sulfation on the activity and selectivity of the hydrocracking products (Figure 2a). Apart from RuSZ_2_ and RuSZ_4_, which achieved 96% and 26% LDPE conversion, respectively, all other catalysts resulted in full conversion at 250°C after 4 h (see Methods for analysis details; typical product GC spectra are shown in Figures S3 and S4). The SZ support alone was inactive for the cracking of LDPE (Table S3), likely because of the high activation barrier (> 500°C) in the absence of a metal species [27]. Non‐sulfated RuZ exhibits a high 76% yield of C_1_‐C_4_ products, with a particularly high content of CH_4_ (46% yield). This indicates that hydrogenolysis is the predominant depolymerization pathway on RuZ, which is typical for monofunctional catalysts [28]. In contrast, the bifunctional RuSZ*_x_* catalysts promote the hydrocracking of LDPE, as revealed by the marked increase in liquid alkane yields (Figure 2a). However, the catalytic activity decreases at high degrees of sulfation, as evidenced by the reduced overall conversion and lower selectivity toward lighter alkanes using RuSZ_2_ and RuSZ_4_ (Figure 2b), which may arise from excessive surface sulfate species hindering hydrogen transfer.

Notably, C_1_ and C_2_ products from the reactions employing RuSZ*_x_* catalysts with a degree of sulfation above *x* = 0.5 M are not observed. The absence of CH_4_ suggests that terminal C─C bond cleavage is highly unfavorable on RuSZ*_x_*, which is unusual for Ru‐based catalysts [15, 16, 17, 18, 19, 20, 21, 22, 23, 24, 25], and is likely linked to the high iso‐/*n‐*alkane ratio produced by these reactions. From the RuSZ_1_‐catalysed reaction, only a combined C_3_ and *n*‐C_4_ yield of ∼7% was present in the gas products, while the remaining 17% was made up of *iso*‐C_4_, representing the smallest hydrocarbon units from the LDPE depolymerization. A higher degree of sulfation correlates to a higher iso‐/*n‐*alkane product ratio, where almost a twenty‐fold increase was obtained upon increasing the degree of sulfation from RuSZ_0.1_ (0.7) to RuSZ_4_ (12.8). As observed from the NH_3_‐TPD profiles, a greater amount of moderate acid sites is generated at higher sulfation levels, correlating well with the higher degree of product isomerization from these catalysts. From GC‐MS analysis, the branched alkanes mostly comprise 2‐methyl isomers (Figure S4), with isomerization occurring at only one end of the alkane. For instance, 2‐methylheptane was the major branched C_8_ species produced over RuSZ_1_, while only trace amounts of more isomerized products, such as 2,5‐dimethylhexane, were detected. This suggests that C─C bond cleavage occurs immediately after methyl‐rearrangement in the polymer.

### 2.3 Optimization of Depolymerization Conditions

**FIGURE 3:**
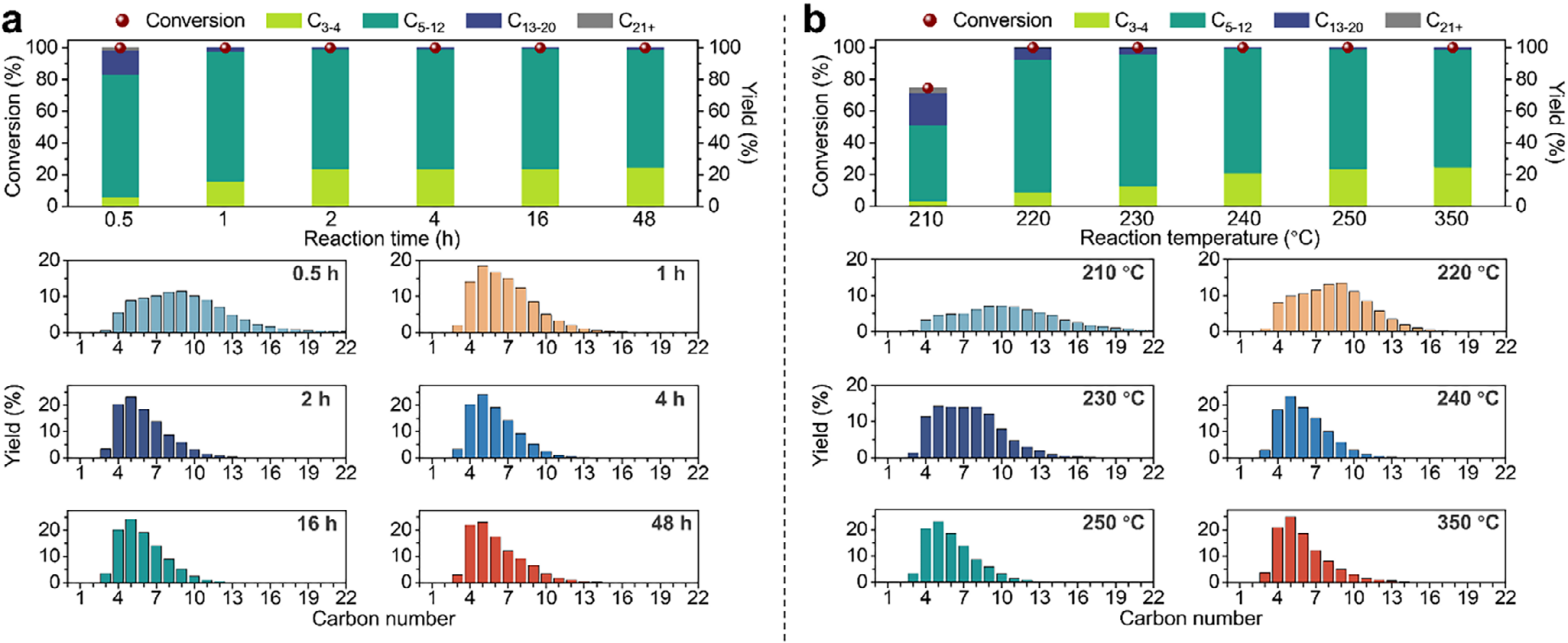
Time and temperature studies. (a) Optimization of reaction time for the conversion of LDPE catalyzed by RuSZ_1_. Reaction conditions: 500 mg LDPE, 50 mg RuSZ_1_ (0.5 wt. % Ru), 250°C, 30 bar H_2_. (b) Optimization of reaction temperature for the conversion of LDPE over RuSZ_1_. Reaction conditions: 500 mg LDPE, 50 mg RuSZ_1_ (0.5 wt. % Ru), 30 bar H_2_, 2 h. Yields of all the products are available in Tables S4 and S5.

Due to its ideal balance between metal and acid sites, RuSZ_1_ was selected for further optimization of the LDPE depolymerization conditions. LDPE was converted at 250°C at different reaction times to investigate the kinetics of the isomerization and hydrocracking processes (Figure 3a). Full conversion of LDPE can be achieved after 0.5 h of reaction, with the product distribution shifting toward heavier liquid alkanes, demonstrating the excellent activity of RuSZ_1_ among Ru catalysts with comparable PE‐to‐Ru mass ratios (200) [15, 16, 17, 20, 23, 25]. Performing the reaction for only 10 min resulted in only 6% conversion (Table S4). These results support a mid‐chain hydrocracking pathway, where the LDPE is rapidly degraded at different segments along the chain into smaller oligomers early into the reaction [5]. The alkane products become lighter with increasing reaction time, up to 2 h, after which the product distribution remains constant, with C_5_ (mainly 2‐methylbutane) at the peak of the distribution (∼24% yield). There was an absence of C_1_ and C_2_ products even after prolonged reaction time up to 48 h, highlighting the unique selectivity of RuSZ_1_. From these results, 2 h was used for further reaction temperature optimization studies.

Next, the effect of reaction temperature on the activity and selectivity of RuSZ_1_ was investigated (Figure 3b). Decreasing the reaction temperature to 200°C resulted in negligible conversion of LDPE (Table S5), as RuSZ_1_ is only partially reduced at this temperature, whereas 74% conversion was achieved at 210°C. In addition, the product distribution broadens at 210°C, favoring heavier alkane products with the distribution peak between C_8_‐C_10_. At 220°C, full LDPE conversion was achieved with an 84% yield of C_5_‐C_12_ products, which are gasoline range hydrocarbons, and thus was selected as the optimal reaction temperature. Further increase of the reaction temperature resulted in a shift toward lighter alkanes (product distribution peak at C_5_), however, only up to 240°C, after which the product distribution remained unchanged. Despite the harsher reaction conditions, the distribution at 350°C is virtually indistinguishable from that at 240°C, signaling that the products are in thermodynamic equilibrium.

To evaluate the reusability of the catalyst, spent RuSZ_1_ was used for another cycle of LDPE conversion. Although there were no visible changes in the Ru‐sites from STEM analysis of the spent catalyst (Figure S5), the spent RuSZ_1_ had negligible activity when directly used without further treatment (Table S6). Furthermore, determination of the Ru content by inductively coupled plasma mass spectrometry (ICP‐MS) of fresh and spent RuSZ_1_ revealed a negligible change in Ru content after the reaction (Table S2), indicating that the Ru did not leach during treatment. Although loss of sulfur content is a typical cause of SZ deactivation [36], elemental analysis revealed negligible change in the sulfur content after the reaction (Table S1). It is possible that more Ru(II) species were generated under the reducing environment, as an increase in Ru─S coordination from 26% to 45% was observed from XPS analysis of the spent RuSZ_1_ catalyst (Figure S6). Notably, the catalytic activity may be restored by treatment of the spent RuSZ_1_ with an equal equivalent of fresh SZ_1_ (see Methods S1 for regeneration procedure), achieving full conversion of LDPE in the subsequent run (Table S6). The product distribution shifts slightly toward heavier alkanes, likely due to the higher acid‐to‐metal site balance after catalyst regeneration. These results suggest that the reduction of sulfate species and loss of acid sites are the primary reasons for catalyst deactivation [44], highlighting the crucial role played by these sites during LDPE depolymerization.

### 2.4 Investigation of Product Selectivity

**FIGURE 4:**
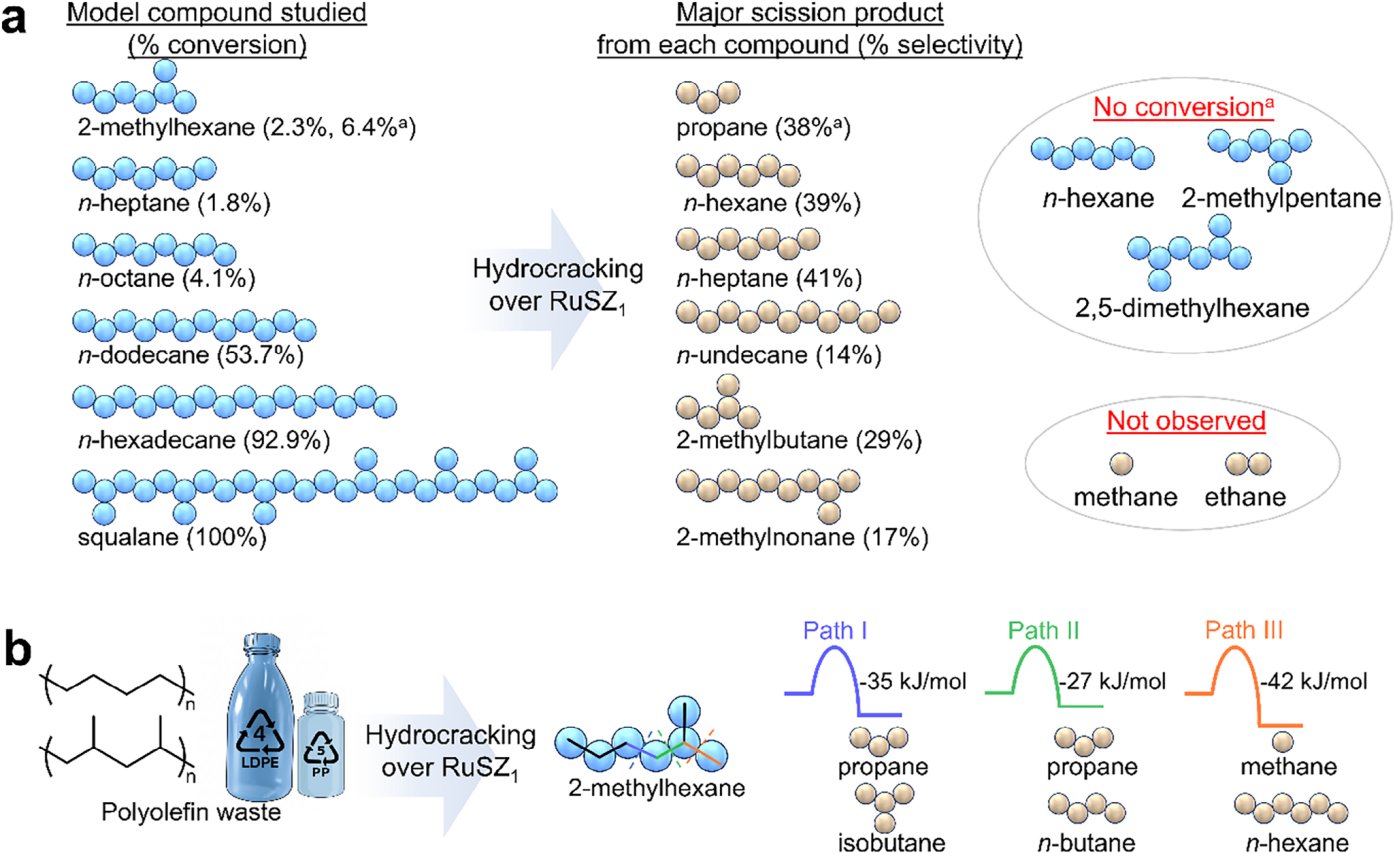
Reaction of simple alkanes in the presence of RuSZ_1_. (a) Alkanes were studied, along with their respective conversions and yields of the major scission products. Reaction conditions: 250 mg alkane, 25 mg RuSZ_1_ (0.5 wt. % Ru), 30 bar H_2_, 250°C, 6 h. (a) Reaction time of 24 h. Yields of all the products are available in Table S7. (b) Calculated reaction energies of different C─C bond cleavage pathways in 2‐methylhexane at 0 K. Reaction energy calculations are shown in Table S8.

To explore the product selectivity of RuSZ_1_, control experiments were performed using various simple alkanes as the reactant (Figure 4a). Interestingly, RuSZ_1_ is inactive toward converting alkanes with fewer than seven carbons, regardless of whether the alkane is linear or branched (*n*‐hexane and 2‐methylpentane). The Brønsted acid sites on SZ can promote alkane isomerization via the generation of a carbenium ion, followed by methyl rearrangement [36], where the C_n<7_ alkanes are less favorable to isomerize due to a high energy barrier toward generating the carbenium ion [45]. The screening studies reveal that increasing the alkane reactant chain length results in higher rates of conversion, with the highest conversions achieved for *n*‐hexadecane (92.9%) and squalane (branched C_32_, 100 %). This may be attributed to the lower scission preference of RuSZ_1_ for short‐chain alkanes, which are also less favorable to undergo C─C bond cleavage [46, 47]. Although the catalyst exhibited some conversion of *n*‐octane to *n*‐heptane, it generated no scission products from 2,5‐dimethylhexane (doubly branched C_8_), suggesting that isomerization precedes C─C bond cleavage following substrate adsorption at the active site. In addition, the formation of C_n‐1_ products from the conversion of C_n_, despite the absence of CH_4_, suggests that alkylation followed by isomerization and cracking of the reaction intermediates occur [36, 48]. Similar to the reactions with LDPE, branched alkanes were detected as the major products at high conversions. However, the conversion of LDPE occurs more readily (full conversion within 0.5 h), as the polymer can likely undergo isomerization at multiple sites before chain scission. These results explain the high consistency of the product distribution observed from LDPE conversion despite differing reaction conditions (1‐48 h, 220°C–350°C), as the major C_7_‐C_12_ products are more susceptible to isomerization than C─C bond cleavage.

To provide a rationale for the observed selectivity, density functional theory (DFT) studies were performed for the conversion of 2‐methylhexane as a representative model of a reactive 2‐methyl species obtained after isomerization and hydrocracking. The free reaction energies at 0 K for three potential C─C bond cleaving pathways were calculated, with Paths I, II, and III shown in Figure 4b. The reaction enthalpies of the pathways are close in energy, from −42 kJ/mol (Path III, most favorable) to −27 kJ/mol (Path II, least favorable), and the energy difference becomes smaller at higher temperatures due to entropic factors (Table S8). DFT simulations were also conducted on the Ru(0001) surface for the three pathways, revealing Path III (hydrogenolysis) to be the most kinetically favorable (Figure S7). The formation of iso‐C_4_ on the Ru surface was found to be highly unfavorable, likely due to steric clashes with the surface in all conformations [49]. However, the absence of methane and prevalence of iso‐C_4_ observed from conversion of alkanes and LDPE suggest that the Ru sites in RuSZ_1_ do not directly facilitate C─C bond cleavage. Instead, these sites likely play an important supporting role by dissociating and transferring H_2_ to hydrocracking intermediates [26]. When Ru was replaced with other hydrogenating metal species (M═Pt, Rh, or Ni), the catalytic activity for the conversion of LDPE was observed to drastically decrease at 250°C (Table S9). RhSZ_1_ and NiSZ_1_ only achieve full conversion of LDPE at 350°C, whereas PtSZ_1_ was inactive. It is noteworthy that the high selectivity to branched C_5‐_C_12_ alkanes remains consistent across all the catalysts, and C_1_‐C_2_ products were not detected.

### 2.5 Conversion of Real‐World Products

**FIGURE 5:**
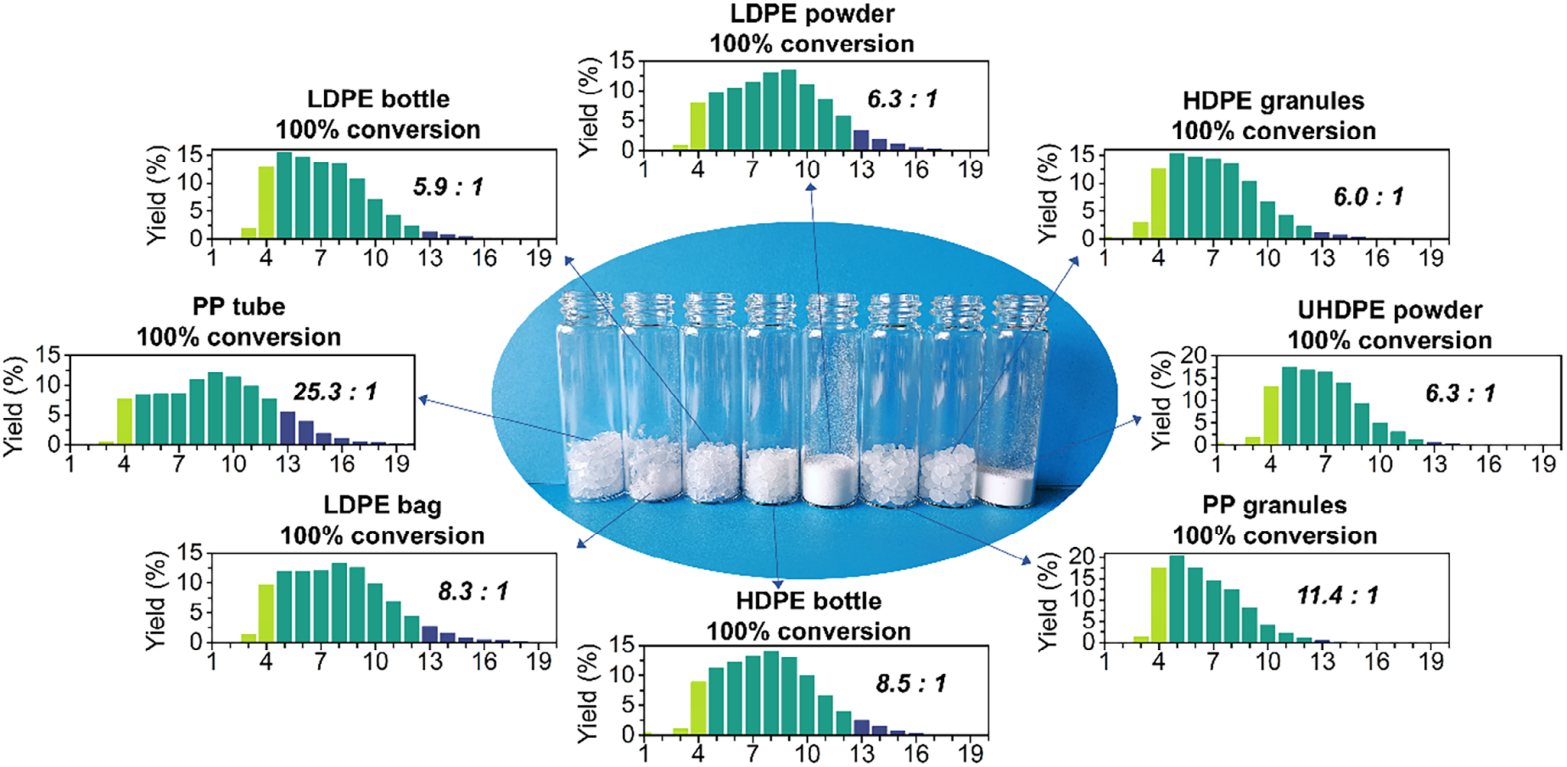
Distribution of alkane selectivities from the conversion of various PE and PP samples catalyzed by RuSZ_1_. The iso‐/*n‐*alkane ratios are shown in italics. Images of commercial polyolefin products (prior to shredding) are shown in Figure S8. Yields of all the products are available in Table S10. Reaction conditions: 500 mg polymer, 50 mg RuSZ_1_ (0.5 wt. % Ru), 220°C, 30 bar H_2_, 4 h (2 h for LDPE powder).

To expand the substrate scope beyond LDPE, multiple PE and PP samples from different sources were investigated for depolymerization using RuSZ_1_ at 220°C. As these polymers have varying M_w_, which may affect the depolymerization rate, 4‐h reactions were conducted to ensure full conversion for all samples (Figure 5). Similar to LDPE, high‐density PE (HDPE granules, M_w_ = 72 400 g/mol) and ultra high‐density PE (UHDPE powder, M_w_ = 6 000 000 g/mol) were converted with an iso/*n‐*alkane ratio of around 6. Despite the significantly higher M_w_ of UHDPE, a similar product distribution was obtained from the conversion of both polymers. This indicates that product selectivity is less dependent on the reaction rate and instead is governed primarily by the scission preference of short‐chain alkanes over RuSZ_1_.

PP (granules, M_w_ 350 000 g/mol) was also fully converted with a higher iso‐/*n‐*alkane ratio of 11.4, due to the high initial degree of branching in the polymer. Based on its lighter product distribution, the conversion of PP occurs more readily than PE. It is possible that the methyl groups in PP stabilize the isomerization intermediates through chain rearrangement, thus promoting hydrocracking [50, 51]. Lastly, experiments were performed on post‐consumer polyolefin products, including an LDPE bag, an LDPE bottle, a HDPE bottle, and a PP tube, which were shredded and directly depolymerized using RuSZ_1_. In all cases, full conversion was achieved, although the alkane distributions slightly shifted toward heavier C_13_‐C_20_ products. These results demonstrate the versatility of RuSZ_1_ as a potential catalyst for waste‐to‐fuel conversion, without the formation of C_1_ and C_2_ gases observed in any case.

To assess the real‐world applicability of the waste‐to‐fuel process, a preliminary cost comparison was conducted between liquefied petroleum gas (LPG, C_3_‐C_4_), gasoline and diesel products obtained via (A) the industrial catalytic hydrocracking of vacuum gas oil (VGO), a by‐product of petroleum refining, and (B) plastic waste processed through the waste‐to‐fuel route (see Methods S3 for detailed information). The analysis indicates that under a mid‐case scenario where the price of plastic waste remains constant (0.40 USD/kg), the cost of gasoline produced from (B) will be 72% less expensive than from (A) (Table S11). In addition, when considering the total cost of producing 1 L each of LPG, gasoline, and diesel, (B) remains 22% less expensive than (A) due to the lower feedstock cost. The similar conditions used for industrial VGO hydrocracking and the present waste‐to‐fuel process enable its potential adoption as an economically incentivized pathway for plastic waste mitigation, particularly if the cost of plastic waste decreases with future improvements in waste sorting.

## 3 Conclusions

We describe a RuSZ_1_ catalyst for the tandem isomerization and hydrocracking of polyolefin waste under solvent‐free conditions. The catalyst exhibits unique selectivity for isomerized liquid alkane products, avoiding C_1_ and C_2_ byproduct formation, irrespective of reaction temperature, duration, or type of polyolefin. Hydrogenolysis on Ru sites is likely suppressed by surface sulfate species, and hydrocracking is expected to occur only at the acid sites. However, metal sites remain essential for H_2_ spillover, as the acid sites exhibit no depolymerization activity on their own at 220°C. Although the acid sites diminish after the reaction, the Ru content remains stable, enabling low‐cost catalyst regeneration. Demonstrated from the conversion of post‐consumer polyolefin products, RuSZ_1_ emerges as a promising catalyst for the efficient valorization of plastic waste to liquid fuels.

## 4 Materials and Methods

### 4.1 Materials

RuCl_3_.3H_2_O was purchased from Precious Metals Online. ZrOCl_2_.8H_2_O, H_2_SO_4_, LDPE powder, HDPE granules, UHDPE powder, and PP granules were purchased from Sigma–Aldrich.

### 4.2 Methods

#### 4.2.1 Synthesis of the SZ_x_ Support

ZrOCl_2_.8H_2_O (16.1 g, 50 mmol) was dissolved in 250 mL ultra‐pure water in a round‐bottom flask. Next, a mixture of aqueous NH_3_ (25%, 15 mL) and ultra‐pure water (25 mL) was added dropwise to the round‐bottom flask until a final pH of 9 was achieved. The resulting mixture was stirred overnight at room temperature, followed by the filtration and washing of the white solid with ultra‐pure water. The resulting Zr(OH)_4_ (∼7.9 g) was dried overnight at 120°C in an oven before sulfation. A total of 1 g of Zr(OH)_4_ was mixed with 5 mL of H_2_SO_4_ (0.1–4.0 M) and kept at room temperature for 2 h. The solid was then collected via centrifugation and dried overnight at 120°C in a vacuum oven, followed by calcination in a tube furnace at 650°C under air flow (heating rate of 5°C/min between 25°C and 300°C and 2°C/min between 300°C and 650°C, and holding time of 3 h at 650°C). Non‐sulfated ZrO_2_ was produced by direct calcination of Zr(OH)_4_. The SZ*_x_* powder (∼ 1 g) was stored in a fumehood before further use.

#### 4.2.2 Synthesis and Characterization of the RuSZ*_x_* Catalysts

The RuSZ*_x_* catalysts were synthesized via the wet‐impregnation method. RuCl_3_.3H_2_O (129 mg, 0.5 mmol, 5 wt. % Ru loading with respect to the support) was dissolved in 5 mL ultra‐pure water and mixed with 500 mg of SZ*_x_*. The mixture was dried overnight in an oven at 110°C, before annealing in a tube furnace at 550°C under air flow (heating rate of 5°C/min and holding time of 5 h at 550°C), to yield the RuSZ_x_ catalysts. RuZ was synthesized with a similar procedure using Zr(OH)_4_ as the support.

Bright‐field TEM images were acquired on a FEI Tecnai electron microscope at 200 keV, and the HR‐STEM and EDX images were acquired on a FEI Themis electron microscope at 300 keV. PXRD of the RuSZ*_x_* catalysts was measured at a voltage of 40 kV and a current of 40 mA on a Bruker D8 Vario diffractometer using Cu Kα (L  =  1.54 Å) radiation. XPS of the catalysts was recorded on a Kratos Analytical Axis Supra spectrometer using the monochromated Kα X‐ray line of an Al anode. The binding energy scale was referenced at 284.8 eV using the aliphatic line of the C 1*s* orbital. H_2_‐TPR of the catalysts was conducted from 30 to 500°C at a heating rate of 10°C /min under a 5.0 vol % H_2_/N_2_ flow, using an Auto Chem II 2910 chemical adsorption instrument. NH_3_‐TPD of the catalysts was analyzed by an Auto Chem II 2910 chemical adsorption instrument. After pretreatment at 300°C for 2 h under a He flow, the samples were saturated with NH_3_ by exposure to a 10 vol % NH_3_/He for 30 min. Subsequently, the NH_3_ desorption profile was recorded from 100 to 650°C at a heating rate of 10°C/min under a continuous He flow.

#### 4.2.3 Conversion of Polyolefins and Product Analysis

To a pre‐weighted 20 mL glass vial, 50 mg of the RuSZ*_x_* catalyst and 500 mg of the reactant were added together with a magnetic stir‐bar. The vial was transferred to a 75 mL stainless‐steel Parr autoclave, which was tightly sealed. The autoclave was pressurized to 20 bar H_2_ and purged to remove residual air, which was repeated for three cycles. The final pressure of the autoclave was set to 30 bar H_2_, and then the autoclave was inserted into a pre‐heated Parr ceramic heating jacket (200°C–350°C) with stirring maintained at 700 rpm. At the end of the reaction, the autoclave was submerged in a water bath under running water for cooling to room temperature.

The autoclave was dried and connected to a Schlenk line for the collection of the gaseous products in a sampling bag. The gas from the sampling bag was injected into a gas chromatogram (Agilent 7000C) with a flame ionization detector (GC‐FID). The spectra of the gaseous products from the conversion of LDPE catalyzed by RuZ and RuSZ_1_ are shown in Figure S3. The yield of gaseous products was calculated from calibration spectra containing known mixtures of (C_1_‐C_4_) alkanes. The liquid products were extracted from the autoclave with diethyl ether, and *p*‐xylene (30 mg) was added as an internal standard, then analyzed on an Agilent 7000C gas chromatogram‐mass spectrometry (GC‐MS) instrument (Figure S4). The liquid products were quantified using the effective carbon number (ECN) method [52], with a *n*‐C_8_—*p*‐xylene calibration curve for absolute quantification. The yields of all alkane products were calculated based on their carbon balance against the initial carbon balance in the reactant.


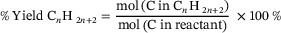




The overall conversion of model alkanes was obtained by directly quantifying the amount of remaining reactant from GC‐MS analysis. The overall conversion of polyolefins was determined by weighing the diethyl‐ether insoluble solid residue, which was assumed to represent unconverted reactant.

$$\begin{array}{ccc} & & \%\;\mathrm{Conversion}\;\mathrm{polyolefin}\;\\ & & \;=\left(1-\frac{\mathrm{Mass}\;\mathrm{of}\;\mathrm{insoluble}\;\mathrm{residue}\;-\;\mathrm{Mass}\;\mathrm{of}\;\mathrm{catalyst})}{\mathrm{Mass}\;\mathrm{of}\;\mathrm{intial}\;\mathrm{polymer}}\right)\;\;\\ & & \;\times \;100\;\% \end{array}$$

## Author Contributions

Xinbang Wu and Sitan Wang contributed equally to the work. Conceptualization: Sitan Wang, Li Shi, Xuan Meng, and Paul J. Dyson. Funding Acquisition: Li Shi and Paul J. Dyson. Investigation: Sitan Wang, Xinbang Wu, Kande Liu, Matilde Onofri, and Kun‐Han Lin. Methodology: Xinbang Wu, Sitan Wang, Kande Liu, Matilde Onofri, and Kun‐Han Lin. Validation: Xinbang Wu and Roland C. Turnell‐Ritson. Supervision: Xuan Meng, Li Shi, and Paul J. Dyson. Visualization: Xinbang Wu, Sitan Wang, and Kun‐Han Lin. Writing – original Draft: Xinbang Wu and Paul J. Dyson. Writing – review and Editing: Xinbang Wu, Roland C. Turnell‐Ritson, Sitan Wang, Xuan Meng, Li Shi, and Paul J. Dyson.

## Funding

This work was supported by the Swiss National Science Foundation and NCCR Catalysis grant number 180544 (X.W. and P.J.D.); the China Scholarship Council (S.W. and L.S.); and the National Science and Technology Council of Taiwan NSTC 113‐2628‐E‐007‐005 (K.‐H. L.).

## Conflicts of Interest

The authors declare no conflict of interest.

## Supporting information




**Supporting File**: advs74215‐sup‐0001‐SuppMat.docx.

## Data Availability

The data that support the findings of this study are available in the supplementary material of this article.
