# Supplementary material for: Selective Conversion of Polyolefin Waste to Branched Alkanes via Methane‐Free Tandem Hydrocracking–Isomerization
Source: Adv Sci (Weinh). 2026 Feb 3;13(21):e16707. doi: 10.1002/advs.202516707 (PMC13073302; doi:10.1002/advs.202516707)
Supplement: Supplementary file 1 — Supporting File: advs74215‐sup‐0001‐SuppMat.docx. [file ADVS-13-e16707-s001.docx]

**Title**

Selective Conversion of Polyolefin Waste to Branched Alkanes via Methane-Free Tandem Hydrocracking–Isomerization

**Authors**

Xinbang Wu^1†^, Sitan Wang^1, 2†^, Matilde Onofri,^1^ Kande Liu^2^, Kun-Han Lin^3^*, Roland C. Turnell-Ritson^1,^ ^4^, Li Shi^2^, Xuan Meng^2^*, Paul J. Dyson^1^*

^†^ Authors contributed equally

*Corresponding authors

Paul J. Dyson ([paul.dyson@epfl.ch](mailto:paul.dyson@epfl.ch))

Xuan Meng ([mengxuan@ecust.edu.cn](mailto:mengxuan@ecust.edu.cn))

Kun-Han Lin ([kunhan.lin@mx.nthu.edu.tw](mailto:kunhan.lin@mx.nthu.edu.tw))

**Affiliations**

1 Institute of Chemical Sciences and Engineering, École Polytechnique Fédérale de Lausanne (EPFL), Lausanne, Switzerland

2 The State Key Laboratory of Chemical Engineering, East China University of Science and Technology, Shanghai, China

3 Department of Chemical Engineering, National Tsing Hua University, Taiwan

4 Chemistry Research Laboratory, Department of Chemistry, University of Oxford, Oxford OX1 3TA, UK

**Table of Contents**

**Supplementary Methods**3

**Supplementary Figures**

S1. Particle size distribution of a) non-sulfated ZrO_2_ b) size of Ru clusters in RuSZ_1_**.**6

S2. HR-STEM analysis of RuSZ_1_7

S3. GC-FID spectra of the gas products from the conversion of LDPE8

S4. GC-MS (FID) spectra of the liquid products from the conversion of LDPE9

S5. STEM-EDX analysis of RuSZ_1_ before and after the conversion of LDPE10

S6. XPS analysis of RuSZ_1_ before and after the conversion of LDPE11

S7. DFT simulations of 2-methylhexane hydrogenolysis on the Ru(0001) surface12

S8. Images of the various PE and PP samples tested13

S9. Process flow diagrams for cost comparison analysis14

**Supplementary Tables**

S1. Sulfur content of fresh RuSZ_x_ and spent RuSZ_1_ catalysts15

S2. Ru content of fresh RuZ and RuSZ_1_ and spent RuSZ_1_16

S3. Conversion and product yields from the reaction of LPDE catalysed by RuZ, RuSZ_x_ and SZ_1_ catalysts17

S4. Conversion and product yields from the reaction of LDPE catalysed by RuSZ_1_ at different reaction times18

S5. Conversion and product yields from the reaction of LDPE catalysed by RuSZ_1_ at different reaction temperatures19

S6. Catalyst regeneration experiments for spent-RuSZ_1_ catalyst20

S7. Conversion and product yields from the reaction of various model alkanes catalysed by RuSZ_1_21

S8. Reaction energy calculations for the conversion of 2-methylhexane22

S9. Conversion of LDPE using other SZ_1_-based catalysts23

S10. Conversion and product yields from the reaction of various polyethylene and polypropylene samples catalysed by RuSZ_1_24

S11. Relative cost of generating 1 L of LPG, gasoline and diesel compared between VGO and PP feedstocks.25

S12. Amount of VGO and PP feedstock required to generate 1 L of respective fuel26

**References27**

**Supplementary Methods**

**S1. Regeneration of RuSZ_1_**

50 mg of fresh RuSZ_1_ was used for the conversion of 500 mg LDPE (see conversion of polyolefins in the Methods section for details). After the reaction, the products and the spent catalyst were dispersed in 10 mL diethyl ether and transferred to a tube for centrifugation. The supernatant was separated from the spent catalyst and mixed with *p*-xylene (30 mg) for GC-MS analysis and product quantification. The spent catalyst was washed twice with diethyl ether and dried overnight at 80 °C before a subsequent reaction without further treatment, showing negligible activity (Supplementary Table S6). To demonstrate catalyst regeneration, another batch of spent RuSZ_1_ was dispersed in diethyl ether with an additional 50 mg of SZ_1_. It was dried for 16 hours at 80 °C and calcinated in the tube furnace at 550 °C in air for 5 hours with a heating rate of 5 °C/min. For the subsequent reaction, the same amount of LDPE (500 mg) was used to keep the metal-to-substrate ratio constant (0.5 wt% Ru) despite an increase in the catalyst mass (~100 mg). The results of the regeneration experiments are shown in Supplementary Table 6.

**S2. Computational details**

The reaction energy profile computations were performed using density functional theory (DFT) with the Perdew–Burke–Ernzerhof (PBE) functional,^1^ incorporating the D3BJ dispersion correction,^2^ as implemented in the Vienna Ab-initio Simulation Package (VASP).^3^ The valence electron wave functions were expanded in plane-wave basis sets with a 400 eV cutoff, and the projector-augmented wave (PAW) method was used to describe core-electron interactions.^4^ A 3 × 3 × 1 k-point grid was sampled using the Monkhorst–Pack scheme for each computation. All structures were fully optimized until the residual forces on the constituent atoms were reduced to less than 0.02 eV Å⁻¹. The climbing image nudged elastic band (CI-NEB) method employed for transition-state searching.^5,6^ The lattice parameter of the primitive Ru was first optimized, where a 6x6 4-layer (0001) slab with a 15 Å vacuum layer was then created. A conformational search for products of three pathways was performed to find the adsorbate configuration with the lowest energy. The energies of these conformers and the transition states were then used to construct the reaction energy diagram.

**S3. Cost comparison analysis of liquid fuel production routes**

Vacuum gas oil to fuels

Vacuum gas oil (VGO) are heavy component hydrocarbons (C_n>20_) distilled from crude oil (15-30% by composition) which are further refined to extract valuable liquid components.^7^ As a major product of crude oil refinery, VGO is traded as a commodity with a market price of $580/t ($0.58/kg).^8^ In the petrochemical industry, VGO is typically processed into light alkanes, including liquefied petroleum gas (LPG, C_3_-C_4_), gasoline (C_5_-C_12_) and diesel (C_13_-C_20_), via hydrocracking over zeolite catalysts at 350-500 °C, under pressures of 150-200 bar and a H_2_/VGO feed ratio of 500-1000 L/L.^9,10^ For a preliminary cost comparison analysis, VGO hydrocracking yields are used for a standard fixed-bed reactor reported by Qader and Hill, where 6.7% LPG, 31.7% diesel and 57.6% gasoline yields are achieved at 500 °C, 103 bar H_2_, space velocity (volumetric flow rate of feed divided by volume of the catalyst bed) of 0.5 h^-1^, over 10 kg Ni-W/zeolite with a H_2_/VGO feed ratio of 500.^9^

Plastic waste to fuels

The conversion of PE and PP plastic waste into liquid fuels using a RuSZ_1_-catalyzed hydrocracking process was demonstrated in this work. For a preliminary cost comparison analysis, the relative cost of LPG, gasoline and diesel is compared using PP waste with VGO in a model reactor. Three scenarios are developed, where the cost of scrap PP varies. Case 1, priced at $0/t, a best-case scenario where the cost of plastic waste collection and separation is not included in the relative cost of products. Case 2, priced at $400//t, mid-case scenario reflecting current market prices of scrap PP.^11^ Case 3, priced at $800/t ($0.80/kg), conservative scenario if the supply of plastic waste decreases in the future due to improved recycling efforts.

Due to the similarities in chemical properties of VGO and PP, identical reaction conditions were used for the conversion of waste PP in the model reactor. The process comprises a fixed-bed reactor at 350 °C, 103 bar H_2_, space velocity of 0.5 h^-1^, employing 600 cm^3^ of RuSZ_1_ with a H_2_/PP feed ratio of 500. In laboratory batch experiments, the H_2_/PP feed ratio is approximately 7 L/L, calculated from (3.75 L of H_2_)/(0.54 L of PP), assuming PP density of 0.92 g/cm^3^. Although the cost of catalysts in both processes can be negated due to possible regeneration, the price of concentrated H_2_SO_4_ ($120/t, $0.12/kg) for regenerating the RuSZ_1_ catalyst is accounted for.

To simplify the cost comparison analysis, some specific assumptions were made (process flow diagram shown in Supplementary Figure S9):

1. The processes are compared in the same reactor (constant CapEx).
2. The cost of energy, water and manpower input in both processes is identical.
3. The amount of hydrogen consumed in both processes is identical.
4. The cost of product distillation, separation and storage in both processes is identical.
5. Each reaction cycle produces 100 L of liquid products.
6. Negligible loss of catalyst after each reaction cycle.

The amount and cost of H_2_SO_4_ needed for catalyst regeneration in each cycle iss calculated as follows:

Amount of SZ_1_ needed in the reactor = 10 kg

Amount of H_2_SO_4_ needed for 1 g of catalyst = $1M\times0.005 L =5 \mathrm{mmol}$

Amount of conc. H_2_SO_4_ (18 M) needed for regeneration per cycle = $\frac{0.005 mol \times10000 g}{18}=2.778 L$ =5.11 kg (assuming density of conc. H_2_SO_4_ to be 1.84 g/cm^3^)

Cost of conc. H_2_SO_4_ needed for regeneration per cycle = $5.11 \times0.12=\$0.61$

Cost of conc. H_2_SO_4_ needed to produce 1 L of products =$\frac{0.61}{100}=\$0.006$1

To calculate the amount of PP required to generate 1 L of each respective fuel (Supplementary Table S11), the yields of PP tube depolymerization from Supplementary Table S10 was used.

**Supplementary Figures**

**
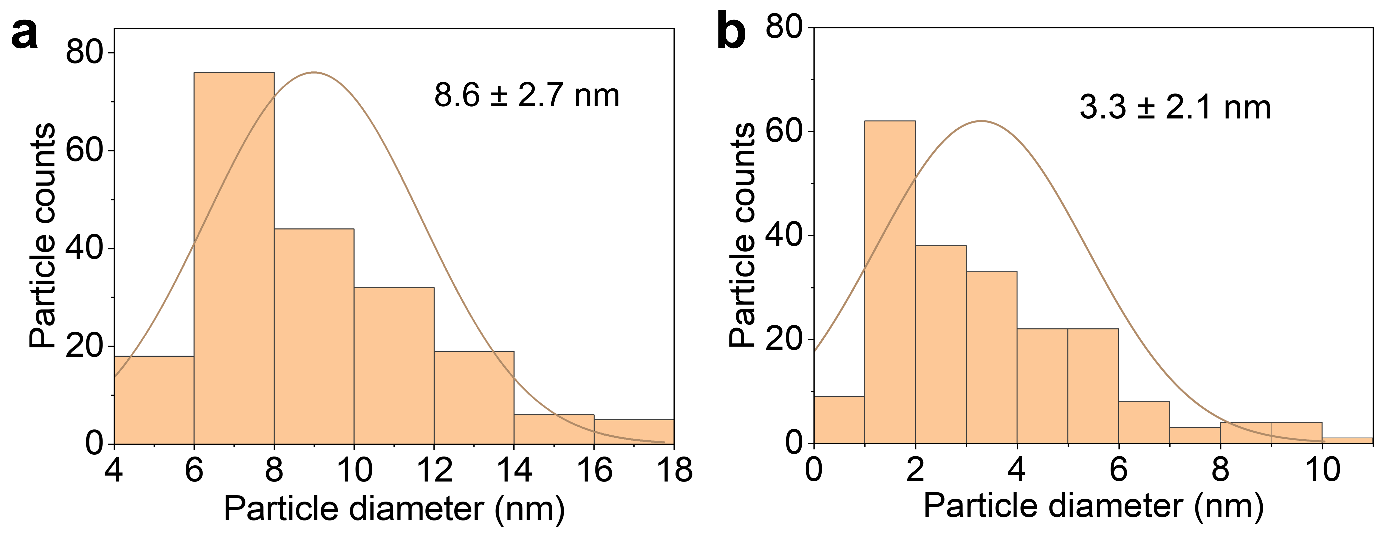
**

**Supplementary Figure S1. Particle size distribution of a) non-sulfated ZrO_2_, and b) the Ru nanoparticles in RuSZ_1_.** The mean size is calculated from the average over 200 particles.


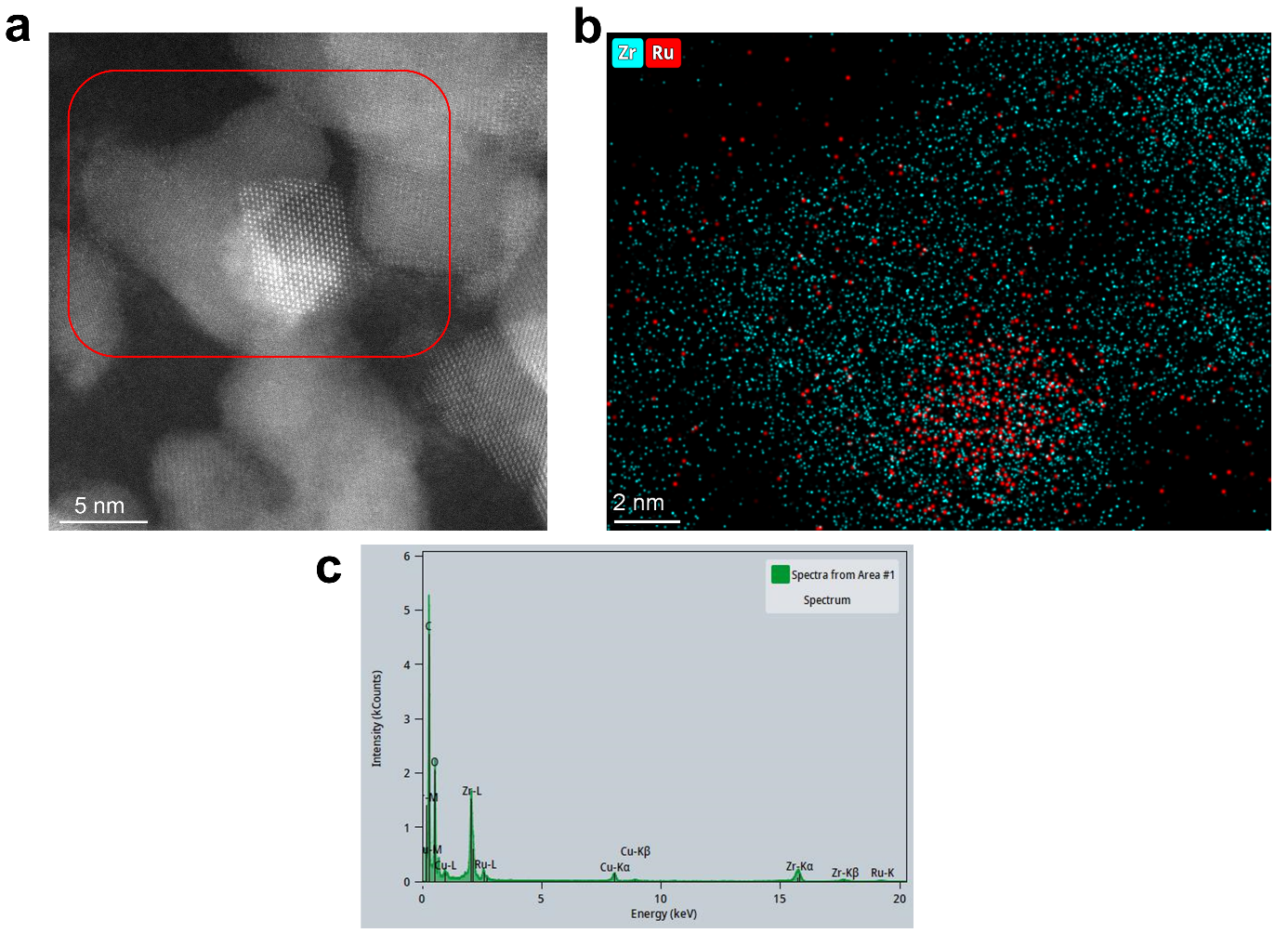


**Supplementary Figure S2. HR-STEM analysis of RuSZ_1_.** a) High-angle annular dark-field (HAADF) image with area marked in red mapped for EDX analysis. b) EDX mapping. c) EDX spectra.


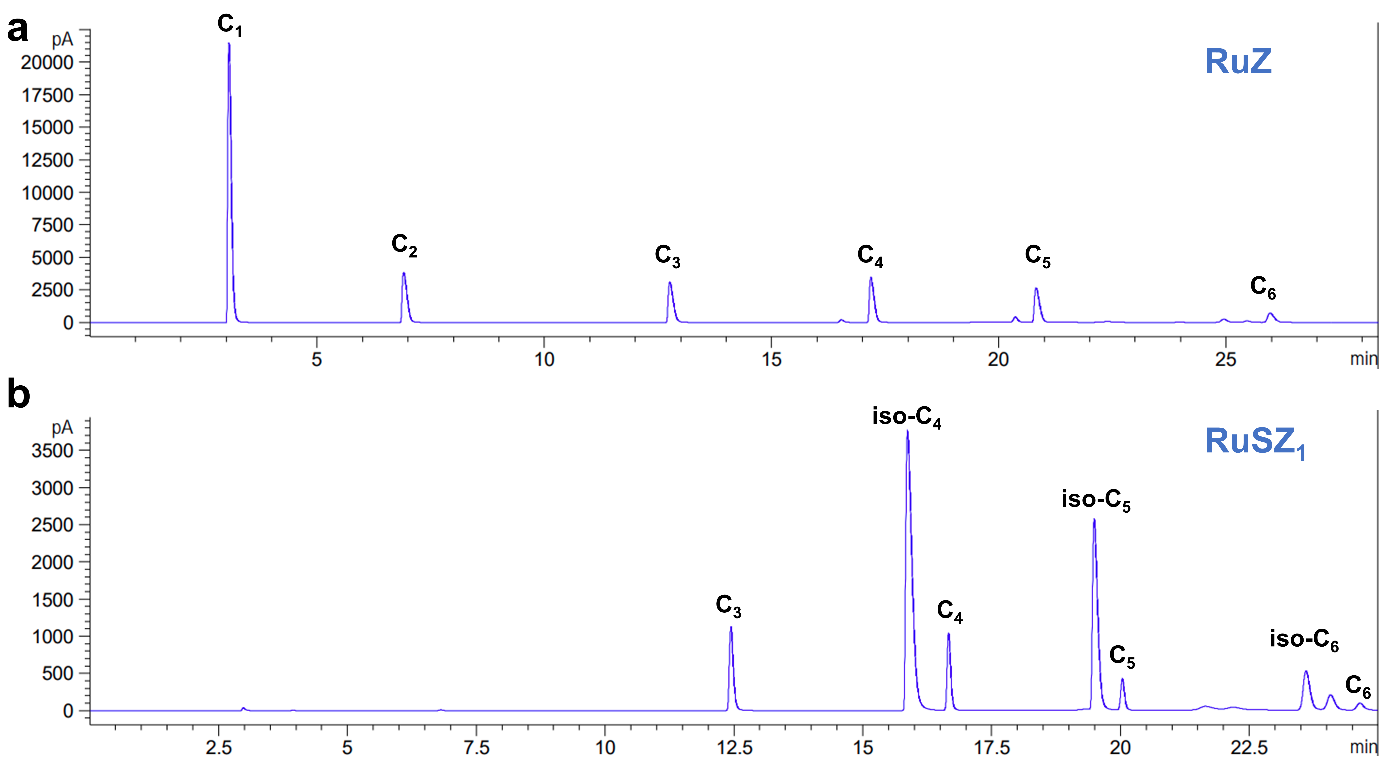


**Supplementary Figure S3. GC-FID spectra of the gas products from the conversion of LDPE catalysed by a) RuZ and b) RuSZ_1_.** Reaction conditions: 500 mg LDPE, 50 mg catalyst (0.5 wt% Ru), 250 °C, 30 bar H_2_, 4 hours. Gaseous samples were injected to identify the retention times of C_1_-C_4_ products and headspace analysis was used to identify the retention times of C_5_-C_6_ products.


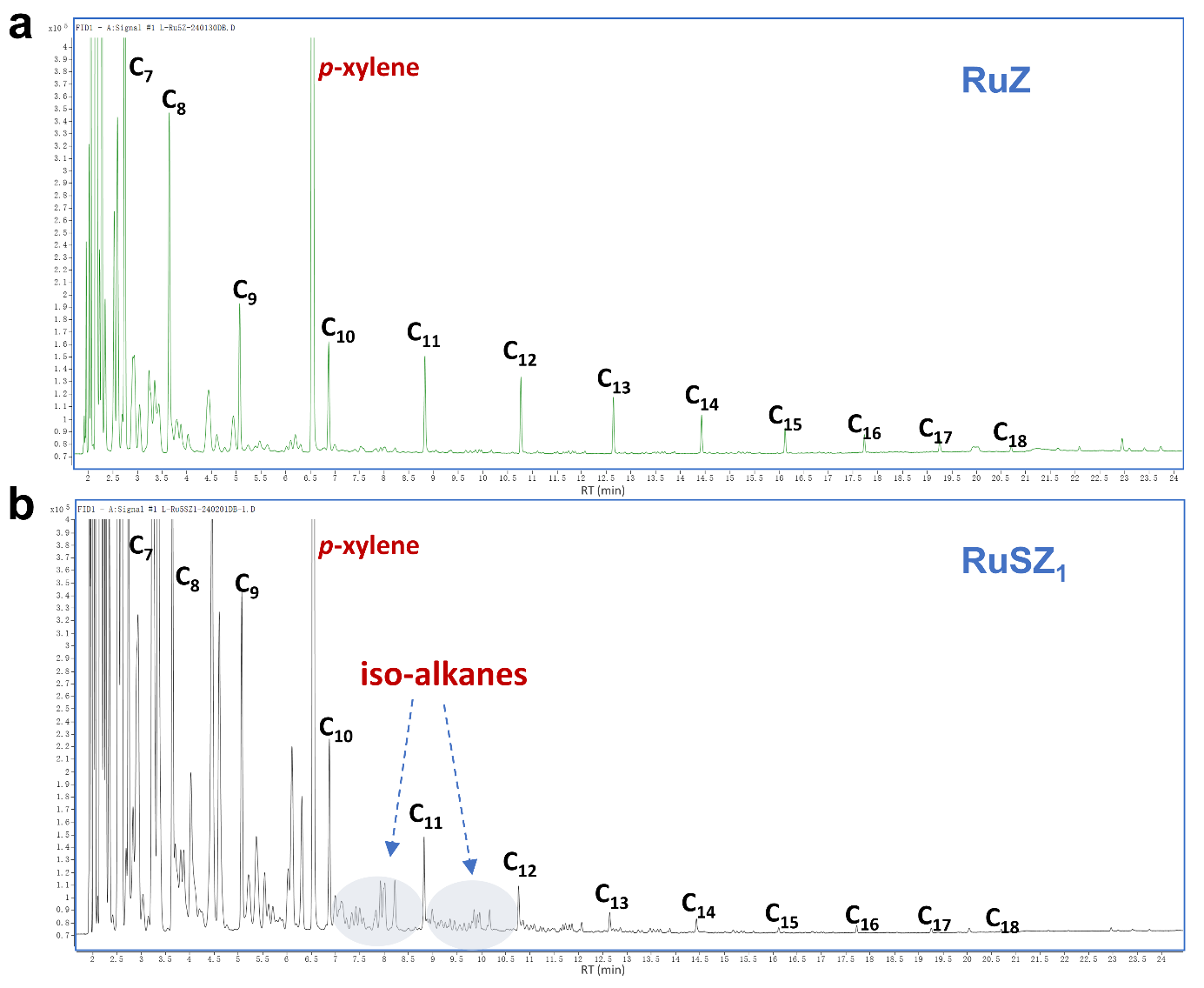


**Supplementary Figure S4. GC-MS (FID) spectra of the liquid products from the conversion of LDPE using a) RuZ and b) RuSZ_1_.** Calibration measurements employing pure *n*-C_6_-C_10_ samples were used to identify the retention times of the *n*-alkanes in this region. A calibration curve was plotted using four *n*-C_8_–*p*-xylene standards for the absolute carbon yield quantification of *n*-C_8_. Isomerized products were identified either via comparison of retention times with pure samples (2-methylpentane, 2-methylhexane, 2-methylheptane and 2,5-dimethylhexane) or from the Agilent NIST library. The quantification of non-*n*-C_8_ products was calculated using the effective carbon number (ECN) method with the following equation:

$$mol of product C_{x} =\frac{\mathrm{mol}\left( nC_{8} \right)\times8}{x}\times\frac{FID area (nC_{8})}{FID area (C_{x})}$$

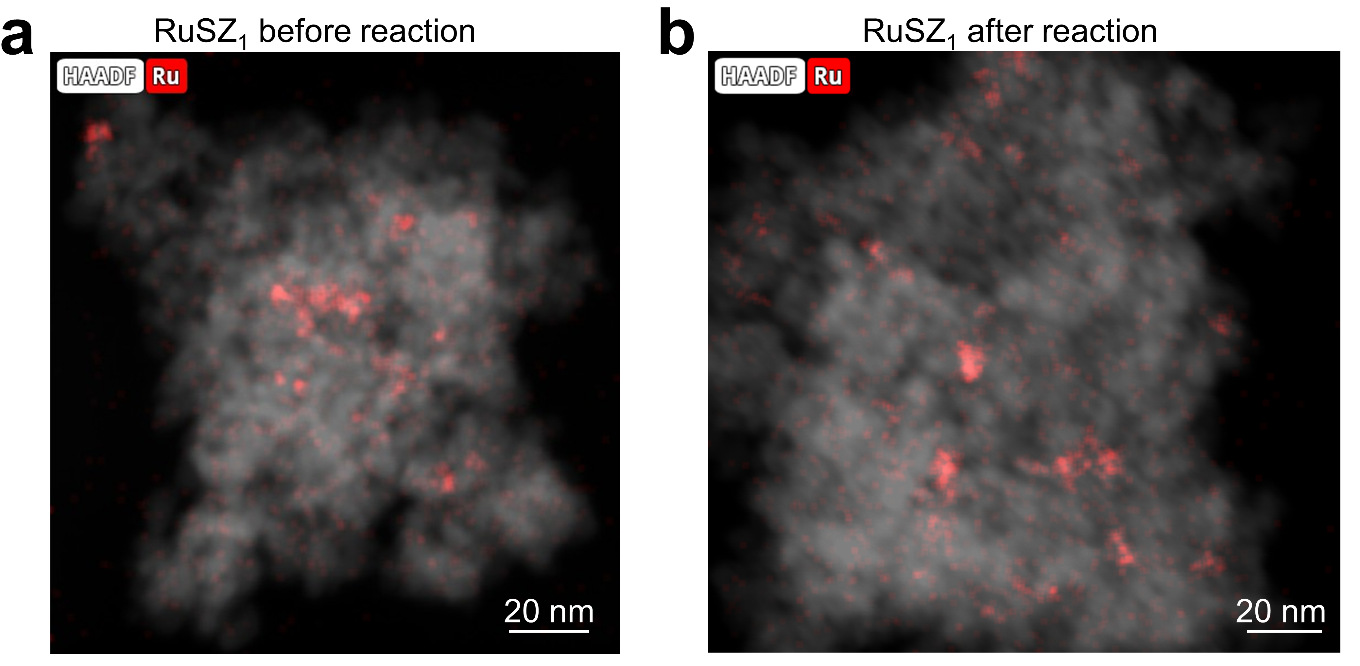


**Supplementary Figure S5. STEM-EDX analysis of RuSZ_1_ a) before and b) after the conversion of LDPE.**

**
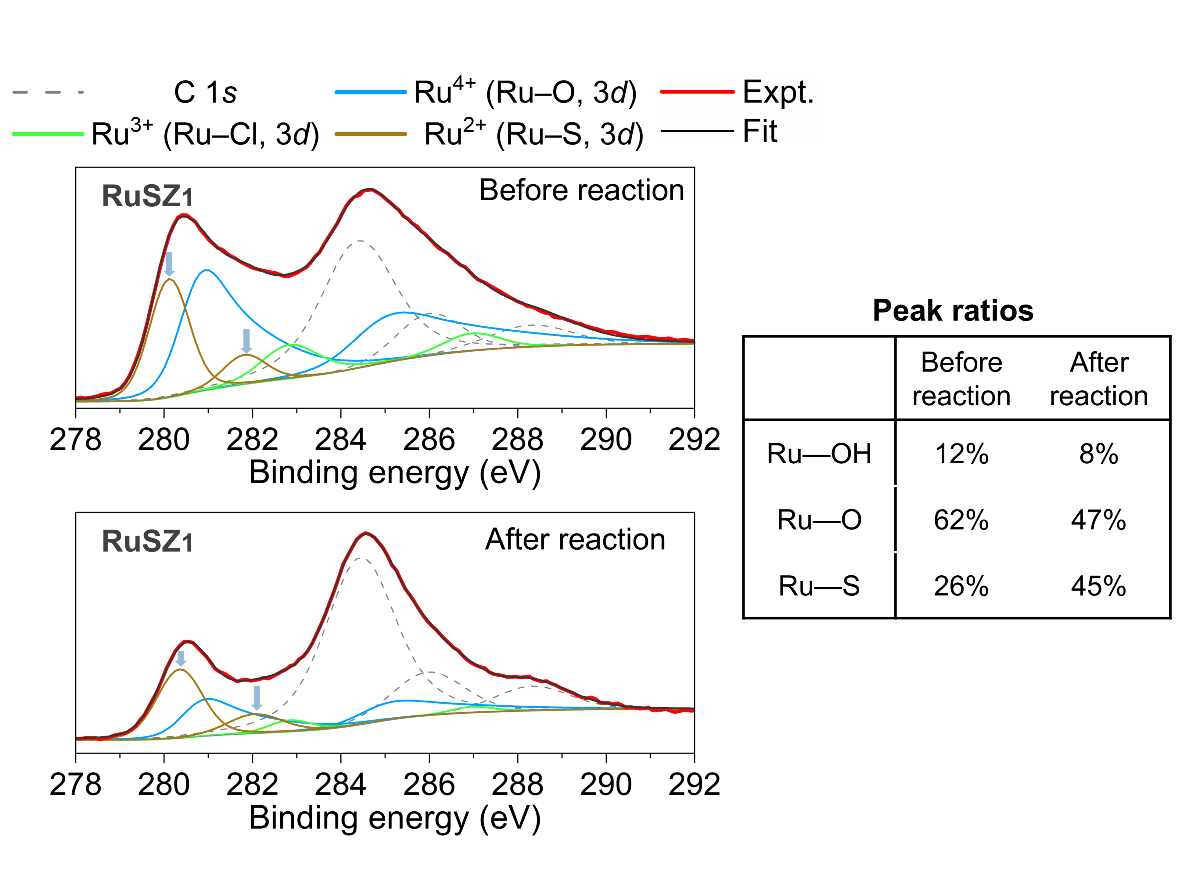
**

**Supplementary Figure S6. XPS analysis of RuSZ_1_ before and after the conversion of LDPE.** The relative ratios of the Ru–OH, Ru–O and Ru–S peak signals are shown on the right. The binding energy of Ru–S shifted from 280.1 and 281.9 to 280.3 and 282.1 eV after the reaction.

**
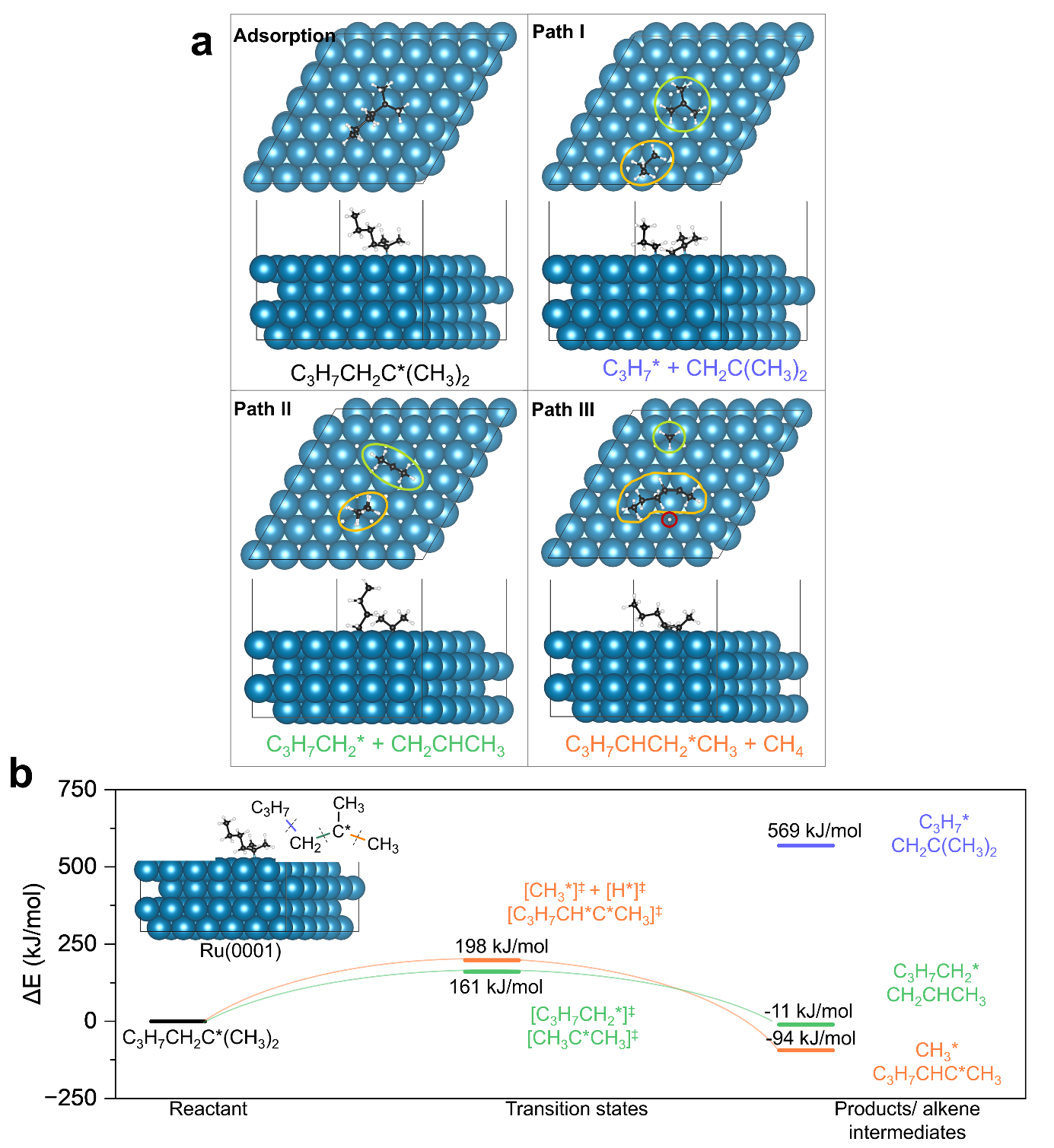
**

**Supplementary Figure S7. DFT simulations of 2-methylhexane hydrogenolysis on a Ru(0001) surface.** a) Conformations of adsorbed reactant on a Ru(0001) surface (top left), and the final conformations of the products after C‒C bond cleavage in each pathway. b) Energy reaction profiles for the conversion of adsorbed 2-methylhexane (C_3_H_7_CH_2_C*(CH_3_)_2_) on Ru(0001) for each pathway. The transition state of Path I was not included due to its excessively high activation energy barrier. Atoms marked with (*) are adsorbed on the metal surface.

**
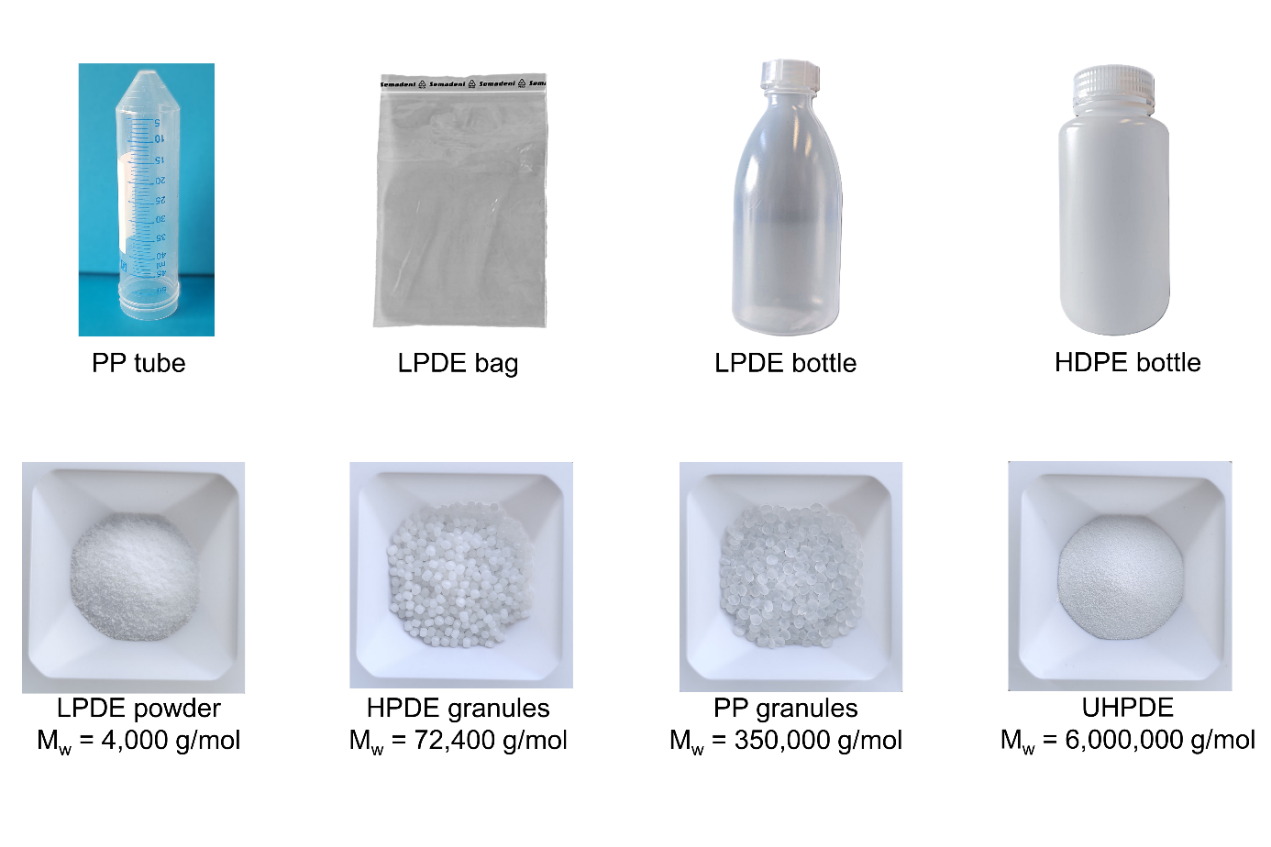
**

**Supplementary Figure S8. Images of the various PE and PP samples tested, including the molecular weight of the pure samples.** The polydispersity index (PDI) of the HDPE and PP granules (isotactic) are 5.7 and 3.5, respectively.

**
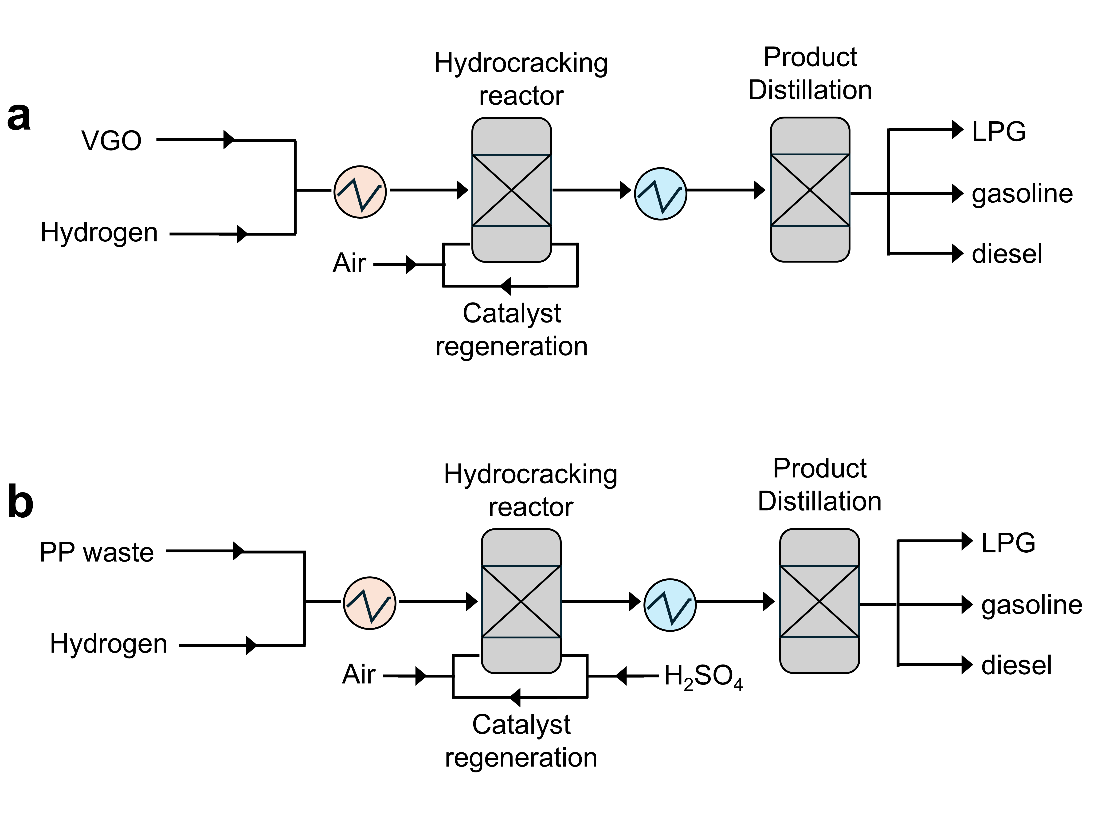
**

**Supplementary Figure S9. Process flow diagrams for the a) VGO and b) plastic waste-to-fuel hydrocracking pathways used for the cost comparison analysis.**

**Supplementary Tables**

**Supplementary Table S1.** **Sulfur content of fresh RuSZ_x_ and spent RuSZ_1_ catalysts.**


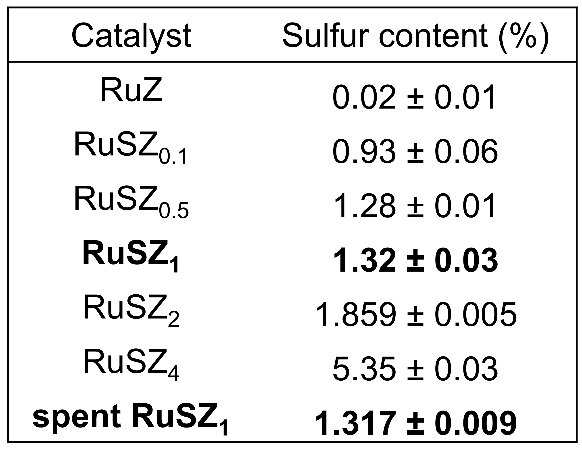


Sulfur content was determined using elemental analysis on an UNICUBE (Elementar) instrument and was calculated from the average of three measurements.

**Supplementary Table S2. Ru content of fresh RuZ and RuSZ_1_ and spent RuSZ_1_.**

**
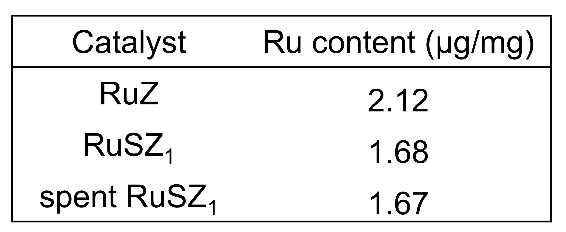
**

Ru content was determined using inductively coupled plasma mass spectrometry on a Nexlon 350 (Perkin Elmer) instrument. ICP-MS analysis of both RuZ and RuSZ_1_ reveals a ~2 wt.% loading of Ru, despite the theoretical loading of 5 wt.%, possibly due to the poor digestion of ZrO_2_ in aqua-regia during measurement. Given the unlikely loss of Ru during the synthesis procedure, a constant 5 wt.% Ru loading was assumed for all the catalysts tested.

**Supplementary Table S3. Conversion and product yields from the reaction of LPDE catalysed by RuZ, RuSZ_x_ and SZ_1_ catalysts.**


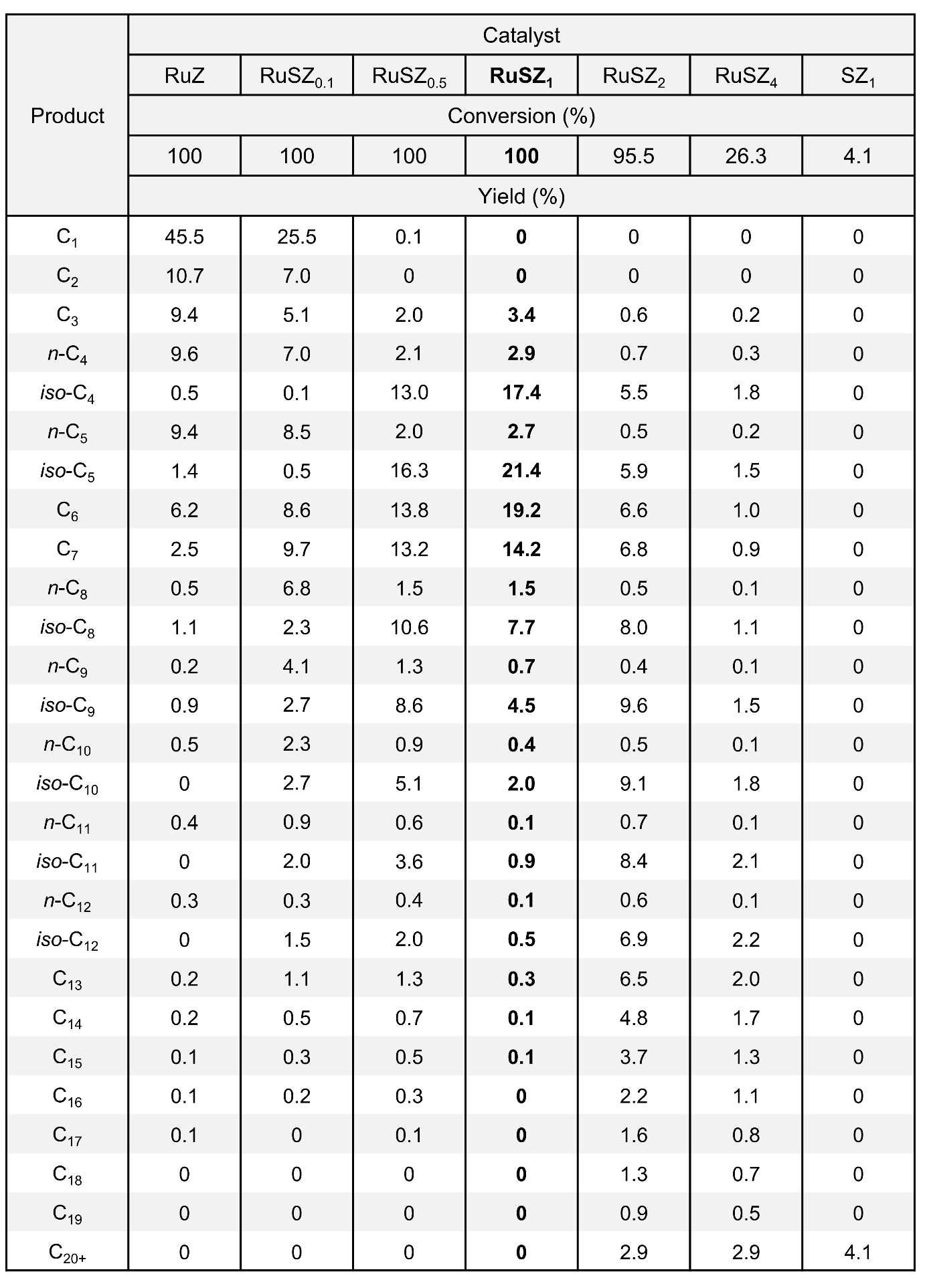


The degree of isomerization of C_6_ and C_7_ products were not determined due to overlap with the solvent peak during GC-MS analysis. Reaction conditions: 500 mg LDPE, 50 mg catalyst (0.5 wt% Ru), 250 °C, 30 bar H_2_, 4 h.

**Supplementary Table S4. Conversion and product yields from the reaction of LDPE catalysed by RuSZ_1_ at different reaction times.**


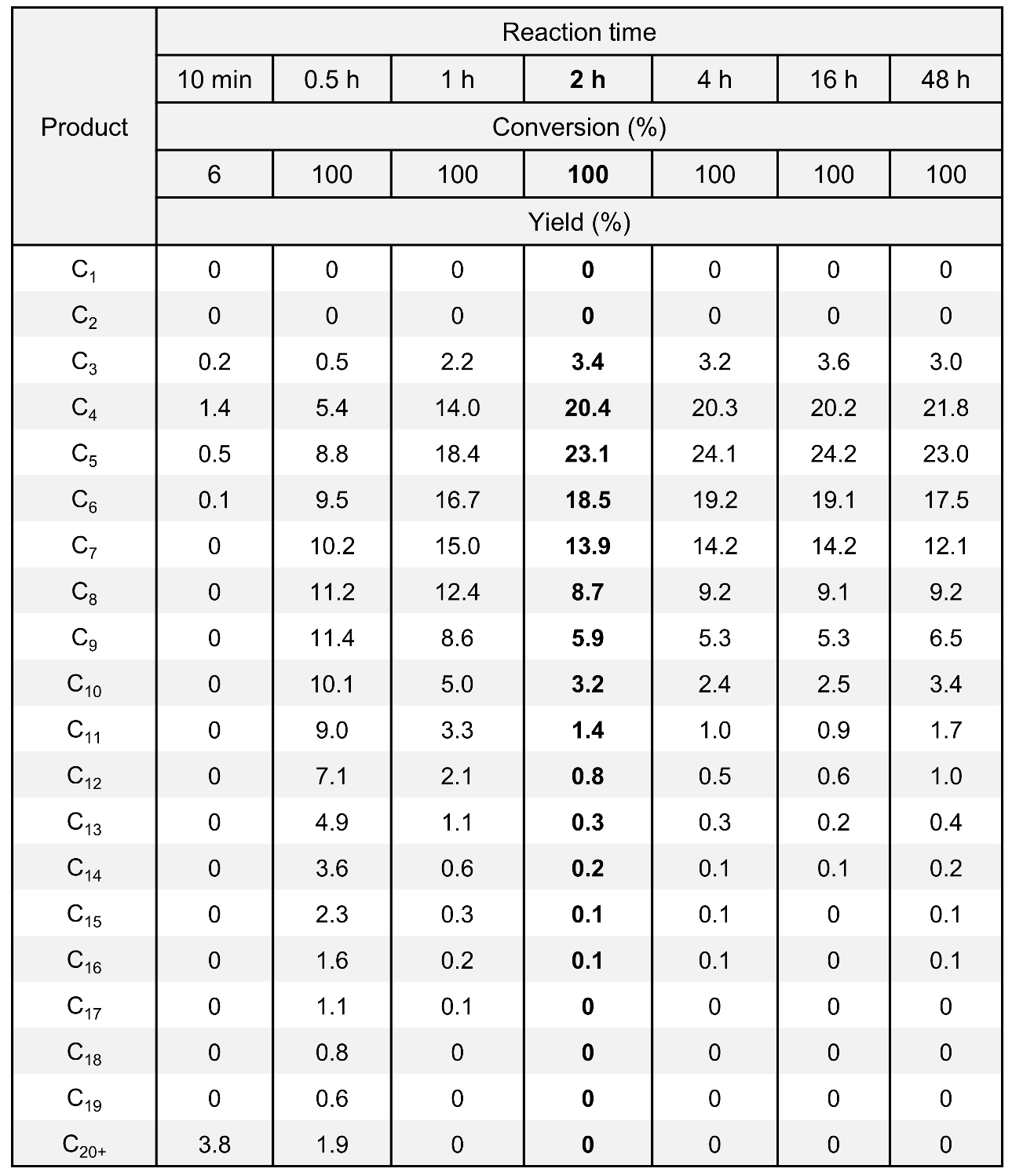


Reaction conditions: 500 mg LDPE, 50 mg RuSZ_1_ (0.5 wt% Ru), 250 °C, 30 bar H_2_.

**Supplementary Table S5. Conversion and product yields from the reaction of LDPE catalysed by RuSZ_1_ at different reaction temperatures.**


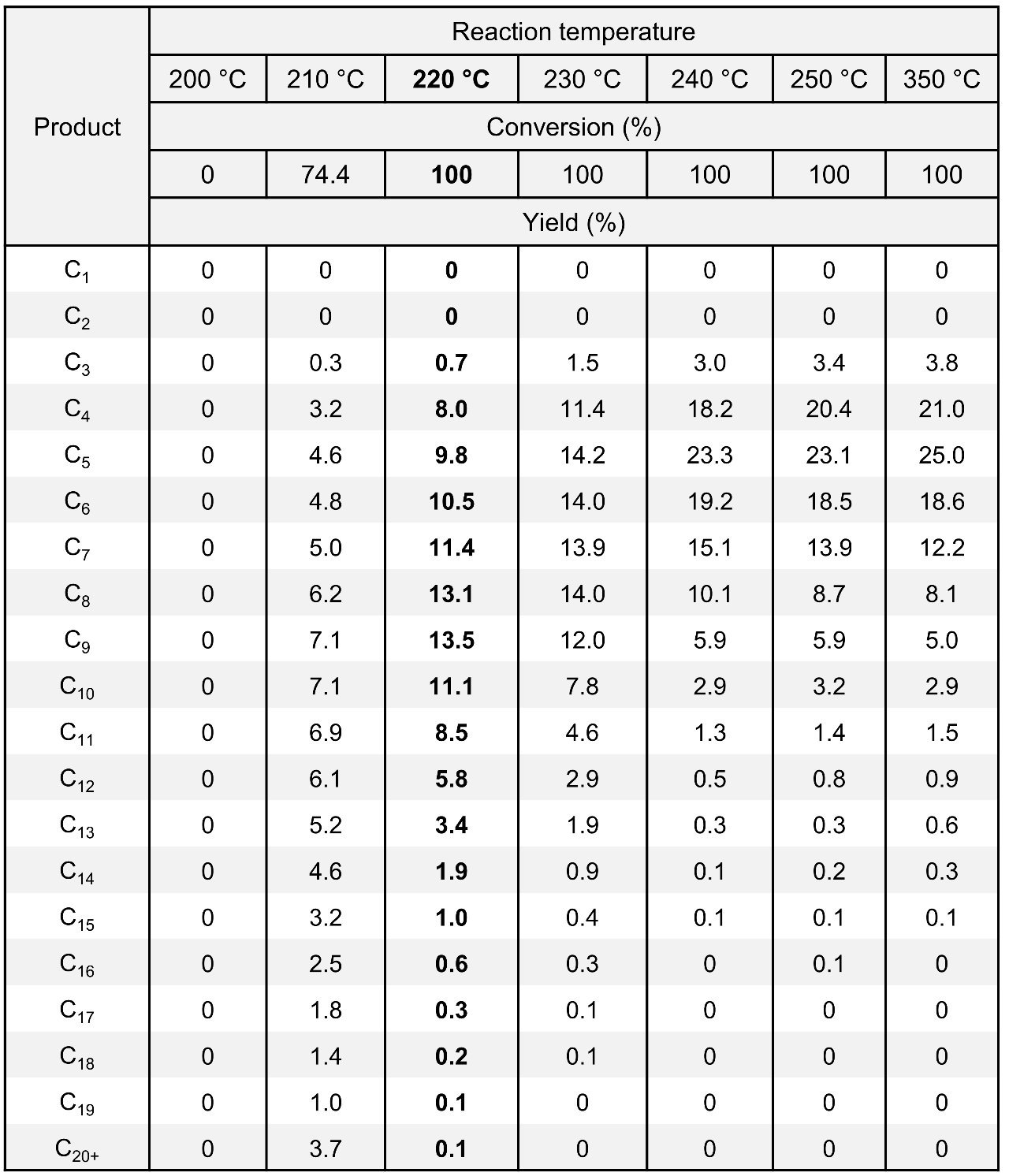


Reaction conditions: 500 mg LDPE, 50 mg RuSZ_1_ (0.5 wt% Ru), 30 bar H_2_, 2 h.

**Supplementary Table S6.** **Catalyst regeneration experiments for spent-RuSZ_1_** **catalyst after conversion of LDPE.**


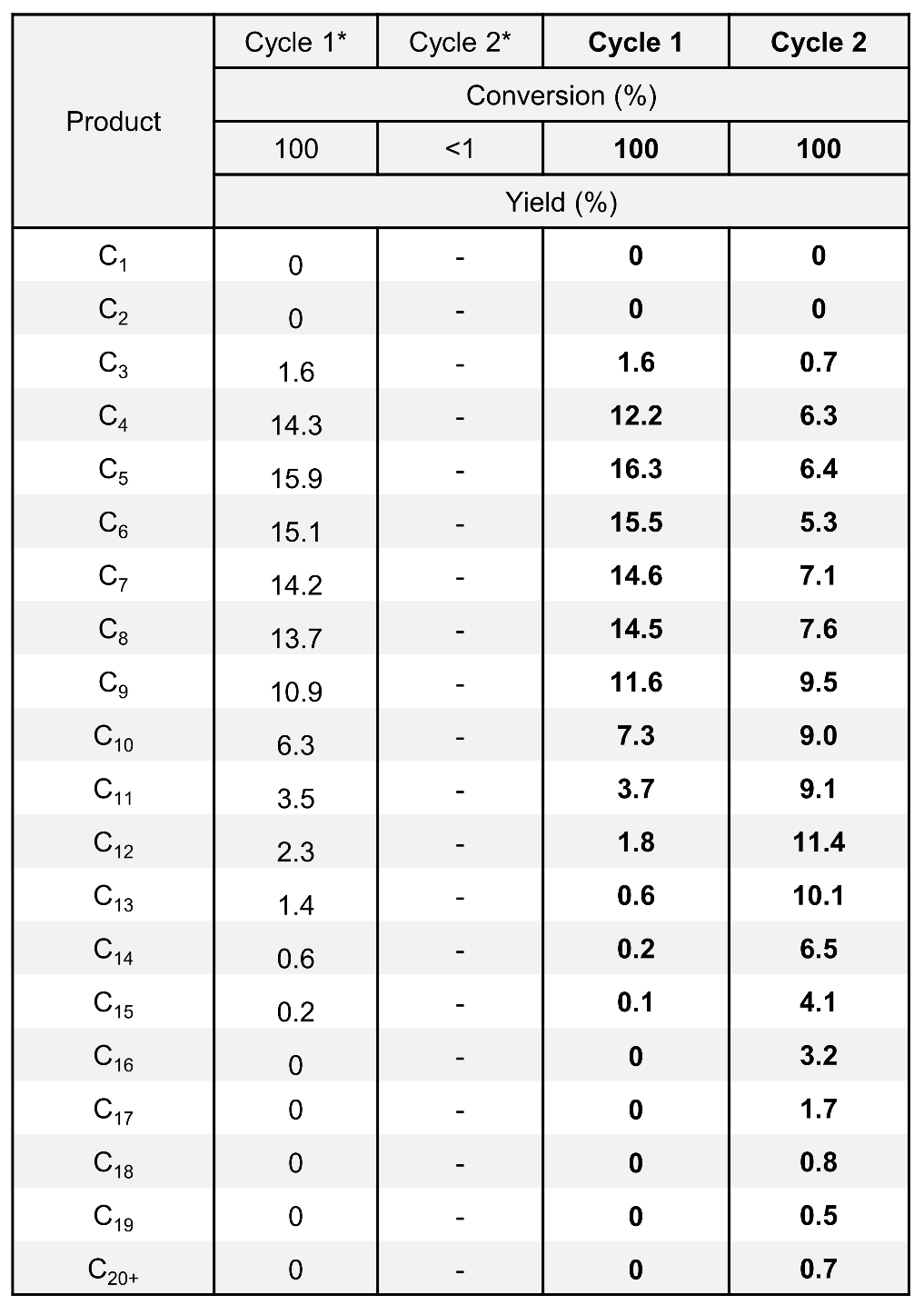


*Without catalyst regeneration. Reaction conditions: 500 mg LDPE, 50 mg RuSZ_1_ (100 mg regenerated catalyst for Cycle 2, 0.5 wt% Ru), 30 bar H_2_, 250 °C, 4 h.

**Supplementary Table S7. Conversion and product yields from the** **reaction of various model alkanes catalysed by RuSZ_1_.**


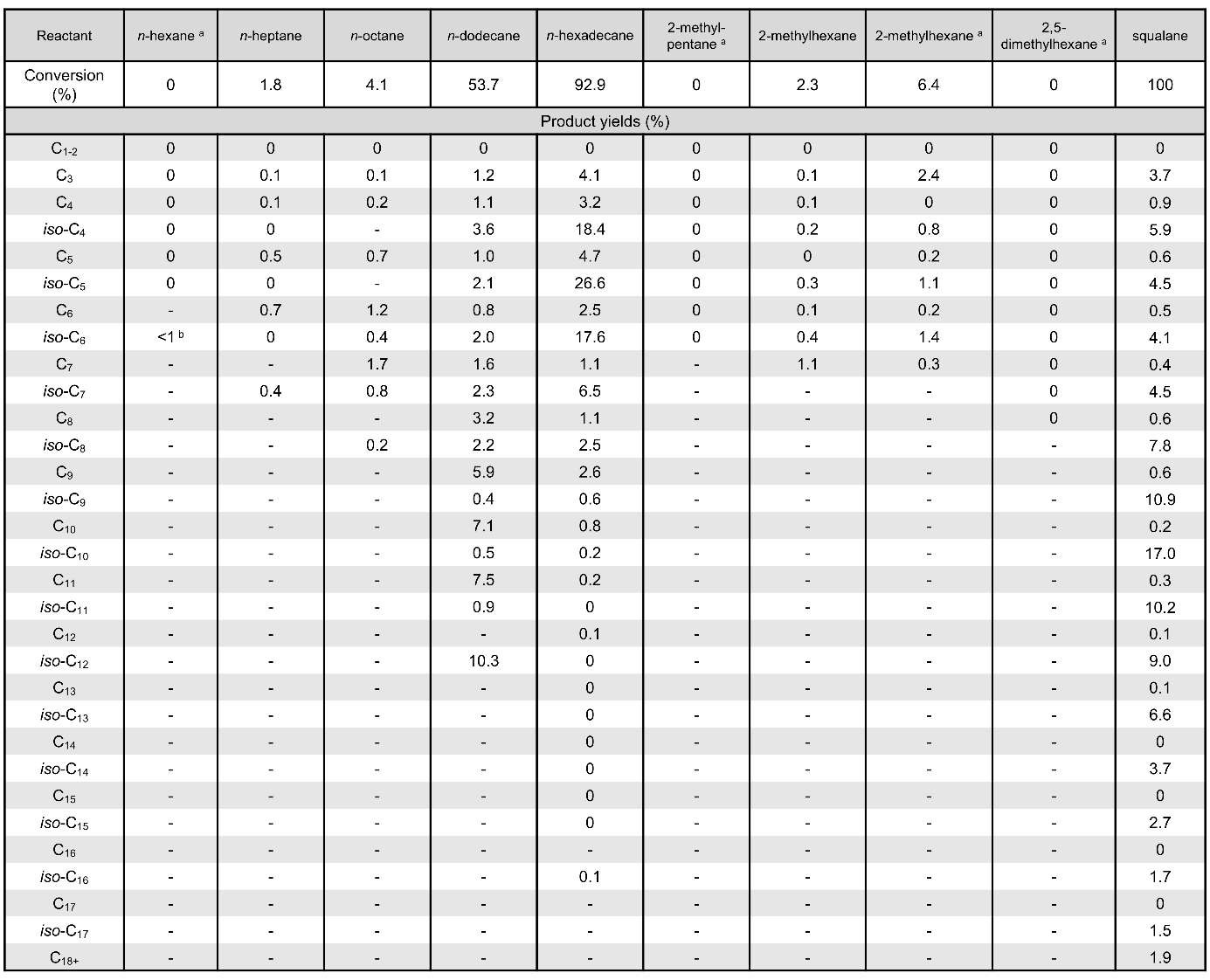


Reaction conditions: 250 mg model alkane, 25 mg RuSZ_1_ (0.5 wt% Ru), 30 bar H_2_, 250 °C, 6 h. ^a^ Reaction time of 24 h. ^b^ Traces of methylcyclopentane detected.

**Supplementary Table S8. Reaction energy calculations for the conversion of 2-methylhexane.**


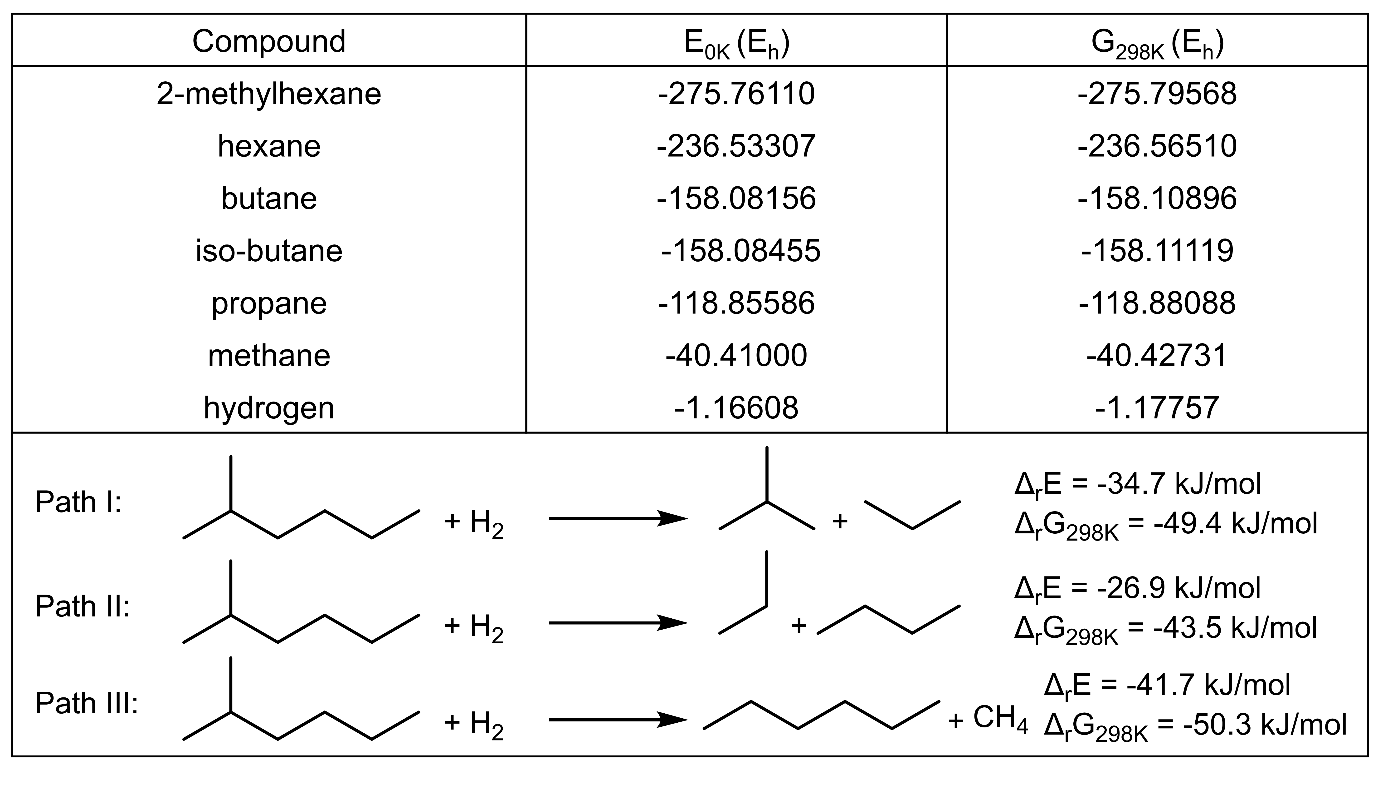


Reaction energy calculations were carried out using the CBS-QB3 composite method.^12,13^ The conversion of 1 E_h_ is approximately 2625.5 kJ/mol.

**Supplementary Table S9. Conversion of LDPE using other SZ_1_-based catalysts.**


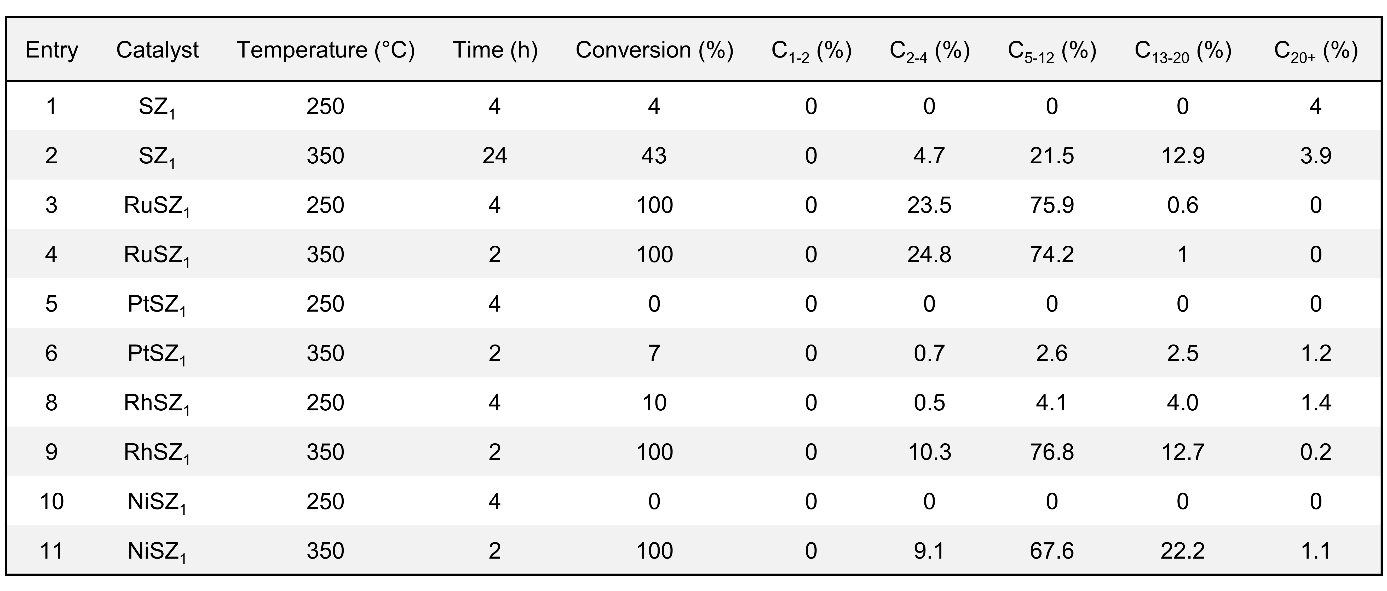


Reaction conditions: 500 mg LDPE, 50 mg catalyst (0.5 wt% metal), 30 bar H_2_.

**Supplementary Table S10.** **Conversion and product yields from the reaction of various polyethylene and polypropylene samples catalysed by RuSZ_1_.**


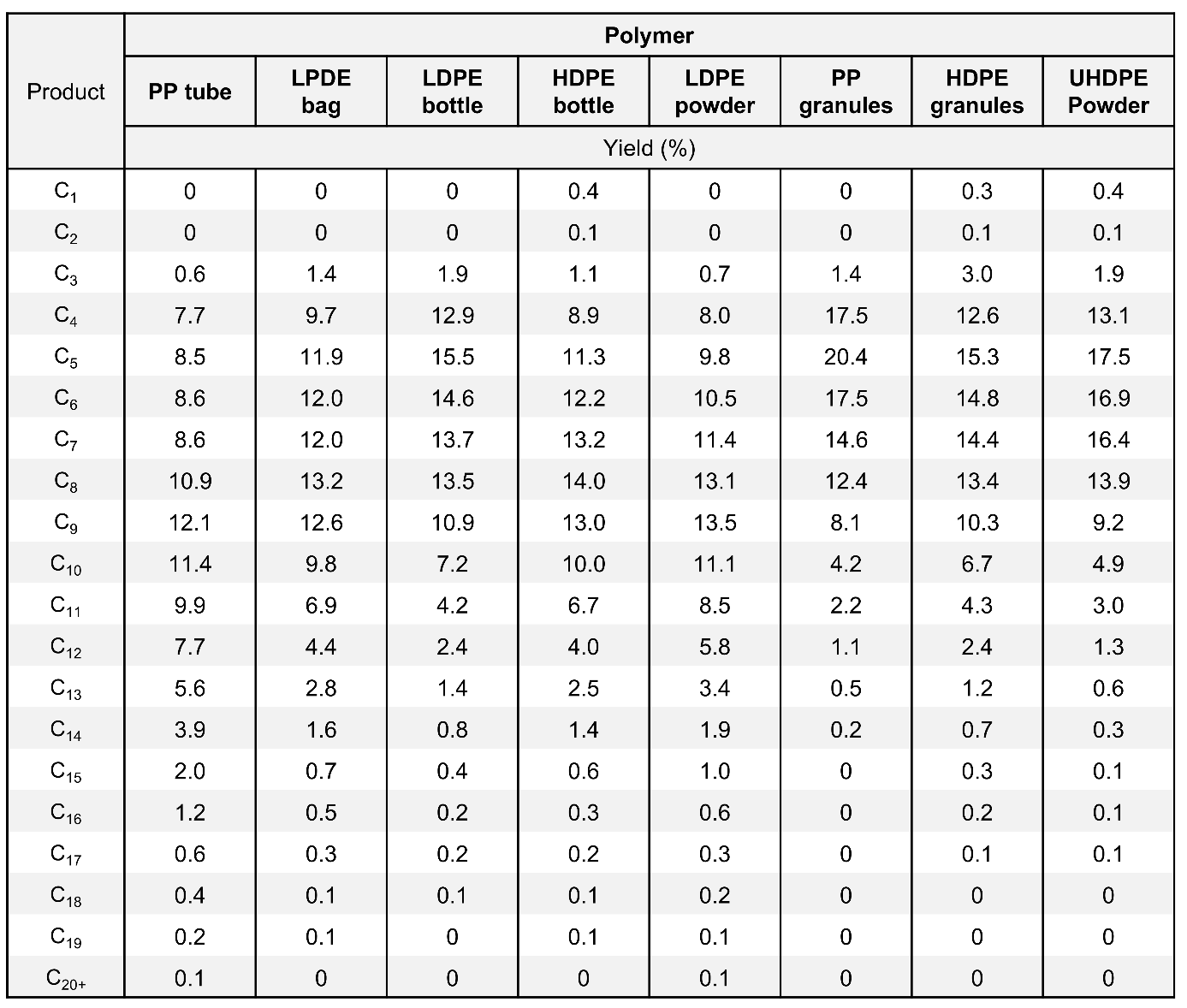


Reaction conditions: 500 mg polymer, 50 mg RuSZ_1_ (0.5 wt% Ru), 220 °C, 30 bar H_2_, 4 h (2 h for LDPE powder).

**Supplementary Table S11. Relative cost of generating 1 L of LPG, gasoline and diesel compared between VGO and PP feedstocks.**

| Product | VGO ($) | PP case 1 ($) | PP case 2 ($) | PP case 3 ($) |
| --- | --- | --- | --- | --- |
| LPG | 4.67 | 0.006 | 2.61 | 5.21 |
| Gasoline | 1.35 | 0.006 | 0.39 | 0.77 |
| Diesel | 0.85 | 0.006 | 2.41 | 4.81 |

The cost of H_2_SO_4_ has been added for the plastic waste-to-fuel processes.

**Supplementary Table S12. Amount of VGO and PP feedstock required to generate 1 L of respective fuel.**

| Product | VGO (kg) | PP (kg) |
| --- | --- | --- |
| LPG | 8.06 | 6.51 |
| Gasoline | 2.33 | 0.95 |
| Diesel | 1.46 | 6.00 |

The density of LPG, gasoline and diesel are assumed to be 0.54, 0.74, and 0.84 kg/L, respectively.

**References**

1. Perdew, J. P., Burke, K. & Ernzerhof, M. Generalized Gradient Approximation Made Simple. *Phys. Rev. Lett.* **77**, 3865–3868 (1996).

2. Grimme, S., Ehrlich, S. & Goerigk, L. Effect of the damping function in dispersion corrected density functional theory. *J. Comput. Chem.* **32**, 1456–1465 (2011).

3. Kresse, G. & Furthmüller, J. Efficient iterative schemes for ab initio total-energy calculations using a plane-wave basis set. *Phys. Rev. B* **54**, 11169–11186 (1996).

4. Kresse, G. & Joubert, D. From ultrasoft pseudopotentials to the projector augmented-wave method. *Phys. Rev. B* **59**, 1758–1775 (1999).

5. Henkelman, G., Uberuaga, B. P. & Jónsson, H. A climbing image nudged elastic band method for finding saddle points and minimum energy paths. *J. Chem. Phys.* **113**, 9901–9904 (2000).

6. Henkelman, G. & Jónsson, H. Improved tangent estimate in the nudged elastic band method for finding minimum energy paths and saddle points. *J. Chem. Phys.* **113**, 9978–9985 (2000).

7. Aitani, A. M. Oil Refining and Products. in *Encyclopedia of Energy* (ed. Cleveland, C. J.) 715–729 (Elsevier, New York, 2004).

8. *Vacuum Gas Oil Market Outlook (2025–2035)*. /www.marketbusinessinsights.com/Vacuum-Gas-Oil-Market. (Accessed 01-01-2026).

9. Qader, S. A. & Hill, G. R. Hydrocracking of Gas Oil. *Ind. Eng. Chem. Process Des. Dev.* **8**, 98–105 (1969).

10. Valavarasu, G. & Sairam, B. Hydrocracking of Vacuum Gas Oil: Conversion, Product Yields, and Product Quality over an Industrial Hydrocracking Catalyst System. *Pet. Sci. Technol.* **31**, 551–562 (2013).

11. Prime prices undermine plastic scrap market. https://recyclinginternational.com/latest-articles/prime-prices-undermine-plastic-scrap/58425 (Accessed 01-01-2026).

12. Montgomery, J. A., Jr., Frisch, M. J., Ochterski, J. W. & Petersson, G. A. A complete basis set model chemistry. VI. Use of density functional geometries and frequencies. *J. Chem. Phys.* **110**, 2822–2827 (1999).

13. Montgomery, J. A., Jr., Frisch, M. J., Ochterski, J. W. & Petersson, G. A. A complete basis set model chemistry. VII. Use of the minimum population localization method. *J. Chem. Phys.* **112**, 6532–6542 (2000).
